# Introducing the Postpartum Toolkit: An Examination of the Feasibility, Acceptability and Pilot Efficacy of an Online Clinical Tool to Enhance Postpartum Functioning and Emotional Wellbeing

**DOI:** 10.3390/jcm11102748

**Published:** 2022-05-12

**Authors:** Ariana M. Albanese, Pamela A. Geller, Jackson M. Steinkamp, Joan R. Bloch, Chris Sikes, Jennifer L. Barkin

**Affiliations:** 1 Department of Psychiatry and Human Behavior, Alpert Medical School, Brown University, Providence, RI 02912, USA; 2 Department of Psychology, Drexel University, Philadelphia, PA 19104, USA; pg27@drexel.edu; 3 Department of Medicine, Hospital of the University of Pennsylvania, Philadelphia, PA 19104, USA; jackson.steinkamp@pennmedicine.upenn.edu; 4 College of Nursing and Health Professionals, Drexel University, Philadelphia, PA 19104, USA; jrb68@drexel.edu; 5 Houston County Health Department, Georgia Department of Public Health, Warner Robins, GA 31088, USA; chris.sikes@dph.ga.gov; 6 Department of Community Medicine, Mercer University School of Medicine, Macon, GA 31207, USA; barkin_jl@mercer.edu

**Keywords:** postpartum functioning, reproductive health, women’s health, mental health

## Abstract

During the postpartum period, a birth parent’s level of functioning (ability to perform the activities and roles required to maintain wellbeing) is critical in determining the health of parents and their infants. However, existing approaches to support postpartum parents are insufficient, especially in the United States, and these individuals face barriers to care. The utilization of internet-based intervention may be an effective solution allowing access to resources for this population. In this study, we developed a patient-centered online tool to bolster postpartum functioning, and collected data on the feasibility, acceptability, and initial impact of this tool on functioning and emotional wellbeing. Data collection took place between February and June 2021 from a sample of 124 individuals who were within the first ten months postpartum and living in the US. Results suggest that the tool is acceptable, though there are barriers to feasibility of use. Additionally, pilot-efficacy data suggest that this tool may be effective in improving postpartum emotional wellbeing, though further controlled testing is warranted. A future iteration of the tool that incorporates participant feedback to improve feasibility of use could prove an effective means of delivering support to an at-risk population.

## 1. Introduction

A birth parent’s level of functioning—their ability to perform the activities and roles required to maintain health and wellbeing—is of high importance in determining parent/child health outcomes during the first postpartum year. Note: we use the term “birth parent” (rather than “mother”) to refer to individuals who are carrying, birthing, and caretaking for their infants. It was taken from a handbook created at Australian National University which suggests the term “birthing/birth parent” should be used as a default, particularly in academic settings, in place of “mother” [1] to promote inclusion and lessen feelings of isolation that can stem from gendered language surrounding childbearing [2]. This is not to suggest that individuals cannot identify as a mother; rather, the assumption of gender should not be made prior to an individual expressing their preferences. This is particularly important as previous work with pregnant transmen has demonstrated that institutional erasure functions as a barrier to routine perinatal care [3]). Birth-parent postpartum functioning is important for determining infant health, as birth parents are generally the principal performers of infant care during the postpartum period, and this is the time in which infants have the greatest need [4,5]. The interaction and care provided to the infant during this time also has implications for later development [6,7]. Postpartum functioning also plays a key role in parent recovery from childbirth, as the postpartum year is a time of increased vulnerability to physical health concerns [8] and is the time period in which individuals assigned female at birth are most likely to develop depression and anxiety compared to any other time in their lives [9]. This vulnerability is so serious that the postpartum period has been determined to have the highest risk of birth-parent and infant death [10]. Therefore, ensuring that birth parents are achieving optimal functioning during this time, and are thus able to address both their own physical and emotional needs, as well as their infant’s needs, is an important health goal.

Importantly, the field has seen recent gains in the measurement and understanding of postpartum functioning. First, with the creation of the Barkin Index of Maternal Functioning (BIMF), the field now possesses a patient-centered means of assessing postpartum functioning across the seven domains of social support, adjustment to new motherhood, infant care, responsibility management, self-care, psychological wellbeing, and mother-child interaction [11,12,13]. Additionally, qualitative work with experts on the postpartum experience, i.e., postpartum birth parents themselves, has revealed key factors that birth parents indicated were most influential in determining their level of functioning during the postpartum year [14].

Despite the recognition that the postpartum year is a vulnerable and important time, existing approaches to support postpartum birth parents are insufficient, especially in the United States [15,16]. Prenatal education, a commonly available initiative, does not appear to consistently prepare birth parents for postpartum adjustment [17,18]. There is also an observed gap between what is desired by birth parents and what is offered by providers during the postpartum period [19,20,21,22], which is an important consideration given that patient-centered approaches have proven influential in delivering effective postpartum mental healthcare [23]. Additionally, a recent review called for better clarification of the dimensions of health that promote holistic birth-parent wellbeing and functioning in the postpartum year, beyond commonly researched topics such as depression and breastfeeding/chestfeeding [24].

Further, postpartum birth parents are a uniquely difficult population to reach. They face many barriers to postpartum healthcare-visit attendance, including the parent reported barrier of being too busy with their new baby and other responsibilities [5,25]. Postpartum birth parents often neglect their own self-care needs, which can reduce postpartum healthcare utilization and engagement in key health-promoting behaviors [26]. Additionally, birth-parent discomfort with disclosing their feelings, often reinforced by their support network’s failure to respond to their needs, can be a barrier to help-seeking behavior [27,28].

Particularly in the realm of mental health care, while some services exist to address postpartum mood and anxiety disorders, screening rates for these disorders are inconsistent and low among healthcare professionals [29] and evidence suggests there are many barriers to addressing mental health in maternal–child health-care settings [30]. This is problematic, as medical providers are positioned to play a key role in connecting patients with mental health care [31]. Research also suggests that birth parents of less-privileged backgrounds, who are at the highest risk for postpartum depression, must contend with attitudinal and instrumental barriers that prevent them from seeking professional mental-health support [32,33,34].

Birth parents in the United States also face systematic barriers, as resources and opportunities such as health insurance, paid parental leave, and nurse home-visiting programs are not universally available [35,36], in addition to a sorely lacking standard of postpartum care compared to “peer” nations. Postpartum health care in the United States generally consists of a single poorly attended six-week postpartum visit, though efforts are being made to move towards a paradigm of more individualized and ongoing care [37]. These obstacles further worsen outcomes for an already at-risk population.

One solution to the issue of resource access for this population is the utilization of mobile health and internet-based intervention. The Pew Research Center has found that internet usage in the US is nearly ubiquitous [38]. A recent meta-analysis found therapist-supported internet-based cognitive behavior therapy to be effective in reducing stress, anxiety, and depressive symptoms among postpartum birth parents [39]. Another recent meta-analysis found social media and mobile health apps to be effective at promoting physical health, mental health, and knowledge during pregnancy and postpartum [40]. Additionally, a recent study that tested the efficacy of a self-guided web-based intervention to improve postpartum mental health, found that this intervention led to significant increases in positive mental-health outcomes [41], demonstrating that interaction with a professional is not necessary in order to confer benefits. Therefore, decreasing barriers to care by allowing for virtual engagement via the internet or a mobile health device shows great promise for delivering support to postpartum birth parents. Additionally, techniques such as individually tailoring virtual interventions have been associated with effective behavior change, and can further increase the impact of such interventions [42].

To address these issues, the current study sought to test the feasibility, acceptability, and pilot efficacy of a newly developed online tool, The Postpartum Toolkit (see Section 2.1 for a detailed description of the tool), based upon qualitative findings uncovered with postpartum birth parents [14]. The Postpartum Toolkit provides postpartum birth parents with personalized resources to bolster functioning in the form of written feedback. Follow-up data was collected six weeks after participant’s received their individual feedback. Feasibility was assessed through participant reports of resource use, and acceptability was assessed through participant ratings of the resource matching the need and usefulness of the resources. Pilot efficacy was assessed by examining pre-post change in score on postpartum functioning (primary outcome), as well emotional wellbeing (stress, anxiety and depression; secondary outcomes).

## 2. Materials and Methods

The study utilized online survey data collection on a secure platform that was designed for this study. IRB approval was obtained by Drexel University.

### 2.1. The Postpartum Toolkit

The personalized resources presented by the tool are determined based off of each participant’s unique response pattern on “The Postpartum Toolkit Checklist” (see Appendix A), which features 24 items representing factors that birth parents identified as most influential to functioning in previous qualitative work [14]. Participants were asked to rate the degree to which each item is a strength for them (selecting from “This is a strength for me”, “This is going ok for me, but could be better” or “I would like help with this”; participant options for responses were based on the Canadian Self-Care Program for Mothers with Postpartum Depression and Anxiety [43]). For each item not endorsed as a strength (i.e., the participant selected “this could be going better for me” or “I would like help with this”), the resources mapped to that item were automatically selected to appear in individualized feedback that was presented to the participant upon submission of the baseline survey. Appendix B includes the text of the resources; each resource is given its own letter (e.g., Resource “H” provides emotion-regulation tools), and Appendix C shows the mapping of resources to checklist items (i.e., which lettered resources would appear for each item on the checklist when not endorsed as a strength). The resources were a mix of information and links to empirically backed educational content. Of note is that efforts were made to include links and recommendations to materials that are low or no cost, not geographically restricted (most commonly online resources), and reflect population diversity. The resources were organized so that the most indicated resources appeared first. Specifically, the resources that corresponded to items on which the participant endorsed “I’d like help with this” appeared before those that corresponded to “This is going ok for me, but could be better.” Of note is that participants were emailed a PDF copy of their feedback for reference and the feedback was calculated to be written at an 8th–9th grade reading level using the Flescher Kincaid criteria [44].

It is important to note that this tool was designed as a flexible and personalized intervention. Depending on the profile of strengths and growth edges of each individual, the tool served different purposes. For some participants, who were indicating areas of growth that called for self-guided intervention (deep-breathing exercises, information about accessing low-cost material resources), this tool could work on its own as an intervention. In some cases, the growth areas endorsed called for resources such as mental-health counseling or specialist care such as postpartum occupational therapy. In these cases, the tool functioned a first step towards getting connected to more intensive support by providing education about services and how to access them.

It is additionally important to note that, prior to pilot testing the tool, we sought consultation from a multidisciplinary group of experts (consisting of: a nurse with expertise in parent/child health, four perinatal-mental-health specialists, an obstetrician-gynecologist, a marriage and family therapist, and two occupational therapists with perinatal expertise) on both the user experience as well as the completeness of the checklist and resource mappings. The experts were presented with documents outlining the content of the tool (see Appendix A, Appendix B, and Appendix C) as well as a description of the user flow. Specifically, they were asked:

1. Whether the checklist of items produced from the qualitative data appear theoretically complete. If it does not appear complete, what items should be added?
2. Whether there are additional resources that should be incorporated into the feedback to address checklist items?
3. What if anything about the way the checklist and feedback is presented (phrasing of information, means of delivering the feedback, etc.) could be improved upon to increase the inclusivity of this tool and/or generally improve the tool?

Feedback from the experts was incorporated into the tool before finalizing it for pilot testing. This feedback included suggestions for more inclusive and sensitive phrasing, more attention to the birth parent’s physical recovery from birth, and adjusting resource offerings to increase accessibility (e.g., including information about accessing doula services for folks of limited financial means).

### 2.2. Participants

Eligible participants were individuals who had given birth in the past ten months (including cisgender females, trans men and non-gender-binary individuals) who live in the United States and had access to the internet. These individuals, additionally, were required to be at least 18 years of age, and both literate and fluent in English, given that all measures were presented in English. Participants also agreed to participate and completed an electronic consent form. Participants were considered ineligible if they were not a cohabitating caretaker for their infant or infants. In an effort to collect feedback from as universal a population as possible, no additional exclusion criteria were imposed.

**Figure 1:**
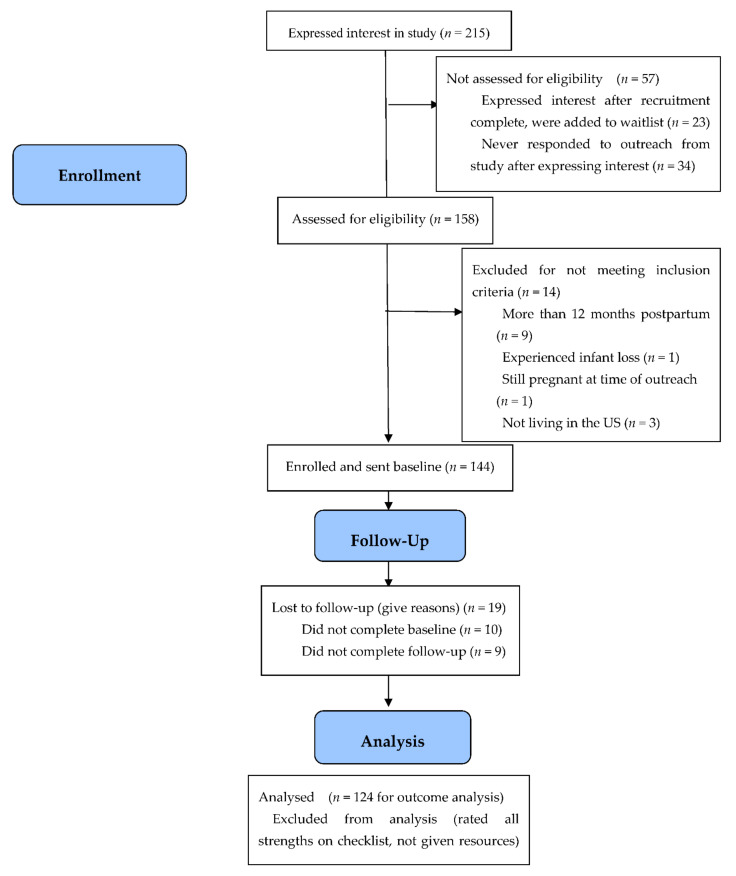
Participant recruitment, enrollment, and flow information.

We planned to recruit 72 completers of the study, as determined by the power analyses. To account for attrition, we planned to enroll up to 150 participants [45]. Ultimately, 125 participants completed all study time points. Overall, 215 individuals expressed interest in the study, 158 of which were assessed for eligibility. Fifty-seven were not assessed for eligibility for the following reasons: 34 never responded to outreach from the study team after they expressed initial interest in participating, and 23 expressed interest after study recruitment was complete. Of the 158 assessed for eligibility, 14 were excluded (9 because they were more than twelve months postpartum, 3 because they were living outside of the US, 1 because they had experienced pregnancy loss, and 1 because they were still pregnant at the time of outreach). Of the 144 individuals who were enrolled, 19 were lost to follow up (10 did not complete the baseline survey, 9 did not complete the follow-up survey). Of note is that 124 individuals were included in the outcome analysis. One individual was excluded from these analyses as they endorsed all strengths on The Postpartum Toolkit Checklist and thus were not presented with resources. See Figure 1 for participant flow information.

### 2.3. Data Collection and Procedure

Recruitment occurred between February and April 2021, and data collection took place between February and June 2021. Participants were recruited online, via postings on online support communities and social-networking websites (e.g., Facebook). Virtual recruitment materials directed interested participants to contact the study team directly or complete the Qualtrics form and a study team member would reach out to interested individuals. Following contact with the study team to confirm that they met inclusion criteria, participants were sent a baseline survey link via email or text (depending upon participant preference) featuring a sociodemographic questionnaire created for the study, the Barkin Index of Maternal Functioning (BIMF) [11], the Perceived Stress Scale 10 (PSS-10) [46], the Hospital Anxiety and Depression Scale (HADS) [47], and The Postpartum Toolkit Checklist, which determined individualized resources provided in participant feedback (as discussed in Section 2.1). Immediately upon submitting the baseline survey, participants were presented with their individualized feedback and resources on the screen of their device (phone or computer). In addition, a study team member checked the data-collection platform daily and emailed PDF versions of the feedback (for reference) to any individuals who had completed the baseline within the past 24 h. Three weeks after the baseline, participants were sent a brief two-item survey by email or text survey inquiring if they have used any of the resources (dichotomous yes or no response), and, if they have used at least one resource, how helpful they found that resource (on a scale 1–10; 10 being most helpful). Six weeks after baseline, participants were sent a final follow-up survey via email or text featuring the BIMF, PSS-10, HADS and a feasibility and acceptability questionnaire created for the study. Participants were asked a series of eleven questions that were created to assess the feasibility and acceptability of The Postpartum Toolkit. Specifically, they were asked:

1. The degree to which the resources that they were given matched their needs (*rated 1–10 with a higher rating indicating a better match*).
2. The degree to which the resources that they were given were found to be useful (*rated 1–10 with a higher rating indicating a higher level of usefulness*).
3. Whether they used the resources (*dichotomous yes/no options for response; if participants responded “yes” a checklist of the resources they were given in their feedback appeared and they were asked to select which resources were used*).
4. Whether there are resources which they have not yet used but intend to use (*dichotomous yes/no options for response; if participants responded “yes” a checklist of the resources they were given in their feedback appeared and they were asked to select which resources have not yet been used but they intend to use*).
5. How often they referenced their feedback (*there were multiple choice options for response*).
6. If they did not use the resources, what got in the way (*there were multiple choice options for response and participants could select all that applied*).
7. Whether there are other factors which should be included in the checklist (*items were listed for participant reference and participants were given a dichotomous yes/no option for response; if they responded “yes” a textbox appeared in which they could specify suggested additions*).
8. Whether there is anything else that could be included in the feedback/resources (*dichotomous yes/no options for response; if they responded “yes” a textbox appeared in which they could specify suggested additions*).
9. Whether there were other resources which the participant used which were not featured in the feedback list (*if they responded “yes” a textbox appeared in which they could specify the resource(s) used*).
10. Whether participants would have been more likely to use these resources if they had a person introducing them to the resources rather than guiding themselves through feedback that was provided automatically (*dichotomous yes/no options for response*).
11. Other suggestions to improve the tool (*dichotomous yes/no options for response; if they responded “yes” indicating they had suggestions, a textbox appeared in which they could specify suggested additions*).

This questionnaire was calculated to be written at a 6th-grade reading level using the Flescher Kincaid criteria [44]. Of note is that individuals who endorsed all checklist items as strengths were not asked to provide feedback on their resources, but were asked for feedback on The Postpartum Toolkit Checklist. After completion of their six-week follow-up survey, participants were compensated with a USD 20 gift card.

### 2.4. Statistical Analyses

Data were analyzed using SPSS 28.0 statistical package. Initial descriptive analyses were conducted to examine the characteristics of the participants and to examine primary variables. Given the generally privileged background of our sample overall, we examined the baseline characteristics of the less-privileged subgroup of the sample to see if the characteristics of this sample seemed to differ from the overall sample. The individuals included in this subgroup were those who filled at least one of the following criteria: were of a non-White racial background, did not possess an advanced degree (individuals whose highest degree was at college or lower), and were of lower income (less than $75,000 estimated annual income given that this was the median US income as of 2018 [48]). In addition, as research on the experience of transgender parents is lacking [49], we examined the data collected from the two participants who did not identify as female (both identified as male). Descriptive statistics were also calculated on the feasibility and acceptability data in addition to qualitative analysis of the open-ended textbox responses on the feasibility and acceptability measure. To assess pilot efficacy, a series of four matched pairs *t*-tests examining mean difference in score from baseline to six-week follow-up were performed on the following variables: the BIMF (primary outcome), the PSS-10, the anxiety subscale score on the HADS, and the depression subscale score on the HADS (secondary outcomes). Assumptions were also tested prior to conducting analyses. For the purposes of statistical evaluation, “completers” were defined as individuals who completed every question in both the baseline and follow-up surveys. Additionally, an exploratory mixed ANOVA was performed to examine if there were significant differences in outcome measures (BIMF, PSS-10, HADS anxiety, HADS depression) between individuals who reported using the resources compared to those who did not report using the resources. In addition, as mentioned, one individual was not included in the outcome analyses as they endorsed all items in The Postpartum Toolkit Checklist as strengths and thus were not presented with resources. Thus, 125 individuals (“completers”) are included in the descriptive statistics of the sample, but 124 are included in outcome analyses.

## 3. Results

### 3.1. Participant Characteristics

Descriptive statistics for all socio-demographics of the sample were examined (see Table 1). Of the 125 participants, the majority of the participants were White (*n* = 102; 81.6%), Non-Hispanic or Latinx (*n* = 115; 92%); identified as female (*n* = 123; 98.4%); were married (*n* = 117, 93.6%); reported access to support for infant care (*n* = 115, 92%); identified as Christian (*n* = 71, 56.8%); possessed an advanced degree (*n* = 70, 56%); were employed (*n* = 102, 81.6%); had an estimated annual income above $75,000 (*n* = 77, 61.6%); and were between the ages of 25 and 35 (*n* = 85, 68%).

Descriptive statistics for participant reproductive and psychiatric history were also calculated (see Table 1). Of the 125 participants, the majority had an infant or infants aged 3–6 months at baseline (*n* = 42, 33.6%), though there was fairly good representation across the first postpartum year. The majority had delivered vaginally (*n* = 89, 71.2%); delivered in their planned mode of delivery (*n* = 111, 88.8%); had given birth to a singleton infant (*n* = 123, 98.4%); had given birth at term (*n* = 119, 95.2%); had a planned pregnancy (*n* = 108, 86.4%); and were primiparous (*n* = 67, 53.6%). The majority of participants were not diagnosed with medical conditions complicating their pregnancy (*n* = 74, 59.2%). The majority of participants also did not have an infant who experienced neonatal intensive care unit (NICU) hospitalization (*n* = 109, 87.2%); had not been re-hospitalized during the postpartum period themselves (*n* = 119, 95.2%); had not received fertility treatment (*n* = 109, 87.2%); and had no history of infant loss (*n* = 91, 72.8%). Lastly, the majority of the sample had a negative psychiatric history (*n* = 87, 69.9%).

#### 3.1.1. Less-Privileged Subgroup Analysis of Baseline Characteristics

It was found that this subgroup was similar to the overall sample in terms of demographics as well as reproductive and psychiatric history. Of the 51 participants in this subgroup, the majority were married (*n* = 45, 88.2%); had access to childcare support (*n* = 47, 92.2%); and were employed (*n* = 38, 74.5%). In terms of reproductive history, the majority had a planned pregnancy (*n* = 41, 80.4%); had an infant 3–6 months old at baseline (*n* = 16, 31.4%); delivered vaginally (*n* = 35, 68.6%); delivered in the mode they had planned (*n* = 45, 88.2%); were primiparous (*n* = 31, 60.8%); had an infant born at term (*n* = 49, 96.1%); did not experience NICU hospitalization (*n* = 44, 86.3%); did not experience a medical condition complicating pregnancy (*n* = 30, 58.8%); did not experience postpartum rehospitalization (*n* = 50, 98%); did not have a history of pregnancy loss (*n* = 40, 78.4%); and did not receive treatment for fertility (*n* = 48, 94.1%). Additionally, the majority had a negative psychiatric history (*n* = 39, 76.5%). However, it was found that that this subgroup was slightly younger than the overall sample, with the majority reporting to be between the ages of 25 and 35 (*n* = 42, 82.4% compared to *n* = 85, 68% of the overall sample) and only 11.8% (*n* = 6) reporting to be in the 35–45 age range (compared to *n* = 34, 27.2% of the overall sample.

#### 3.1.2. Male-Identifying Subgroup Analysis

With regard to sociodemographic factors for this subsample, they were similar to the majority of the sample in most respects (identified as White, were between the ages of 25 and 35, had a college degree, were employed, married, and of above average income). However, with regard to reproductive history, they differed from the majority of the sample. While the majority of the sample (87.2%) did not experience NICU hospitalization, both of these participants’ newborns spent time in the NICU.

### 3.2. Feasibility of The Postpartum Toolkit

Descriptive statistics were calculated on both the mid-study report of resource utilization as well as the follow-up survey report of resources utilization (see Table 2). At the midpoint, 69 participants (55.6% of the sample) reported having utilized at least one resource that was provided in their individualized feedback generated by The Postpartum Toolkit. At follow-up, 70 participants (56.5% of the sample) reported having utilized at least one of their personalized resources. Additionally, at follow-up, most participants reported referencing their resources “a few times” (*n* = 56, 45.2).

The most-commonly endorsed barrier to resource utilization was insufficient time (*n* = 50, 40.32%), followed by forgetting about the resources (*n* = 42, 33.87%). When asked if they would have been more likely to engage with the resources if they had been guided through them as opposed to guiding themselves through the resources, most participants (*n* = 72, 58.1%) reported that they would have preferred a guide.

Table A1 (see Appendix D) displays the number of participants who were recommended each of the 28 possible resources, as well the number of participants who reported that they had either used or intend to use the resource. The most-commonly recommended resources were “Resource 5: Consider seeking professional mental health support” and “Resource 8: Get some distance from your thoughts”, which were both included in the personalized feedback of 122 participants (98.4%). It should be noted that both of these resources were mapped to multiple checklist items (which is not the case for all resources; see Appendix C for resource-to-checklist item mapping), which increased their likelihood of recommendation. The resource with the highest percentage of reported use or intended use by those who were recommended it was “Resource 26: Take care of yourself”, which was included in the feedback of 98 participants (79.03%) and was reported used or intend to use by 44 of the participants (44.9%) to whom it was recommended. The least-recommended resource was “Resource 20: Learn about the resources around you,” which was included in the feedback of five participants. This resource, alongside “Resource 14: Learn about baby care” (recommended to six participants) were the-least used resources. Both were reported used or intend to use by zero participants. The average percentage of use was 24.29%, meaning that the average percentage of individuals who reported that they had used or intend to use a resource was about a quarter of the individuals to whom the resource was recommended.

Relatedly, Table A2 (see Appendix E) shows the frequency counts of responses to The Postpartum Toolkit Checklist items. The item with the highest percentage of participants who endorsed it as an area not requiring extra support was item seven: “I am able to get the materials I need to take care of the baby and myself (such as diapers and food)” (96% reported it as a strength). This was followed by item eight: “I am getting more experienced with taking care of my baby” (95.2% reported it as a strength), and item 15: “My home environment is safe and stable” (93.6% endorsed this as a strength). The items with the least percentage of participants endorsing it as a strength were item two: “When trying new things, I do all of the following: I am patient with myself and don’t expect myself to get things right away, I feel comfortable pushing myself beyond my comfort zone, I can be flexible with my plans and expectations”, and item 16: “I know that taking care of myself is important, and guilt does not get in the way of taking care of my own needs” (21.6% of the sample endorsed each of these as a strength). This was followed by item 13: “I am involved in activities outside of the house (such as friendships, hobbies, professional or volunteer work)” (26.4% endorsed this as a strength).

#### Less-Privileged Subgroup Analysis of the Feasibility of The Postpartum Toolkit

The less-privileged subgroup (*n* = 51) was found to be similar to the overall sam-ple, with the majority of participants reporting that they used the resources (*n* = 28, 54.9%), and referencing their resources “a few times” (*n* = 24, 47.1%). However, a higher percentage of this subgroup would have preferred having a person introduce resources to them rather than guiding themselves (*n* = 35, 68.6% of the subsample compared to *n* = 72, 58.1% of the overall sample). Additionally, like the overall sample, this subgroup reported that the most frequent obstacle to use was not having enough time (*n* = 23, 54.1%), though the percentage of individuals in the subgroup endorsing this was higher than the overall sample (overall sample: *n* = 50, 40.32%).

### 3.3. Acceptability of the Postpartum Toolkit

Descriptive statistics were calculated on both the mid-study report of resource helpfulness as well as the follow-up survey report of resources utilization (see Table 2 for follow-up results). At the midpoint, the average helpfulness rating (on a 1–10 scale with 10 being most helpful) was 7.35 (*SD* = 1.62). At follow-up, the average 1–10 rating of the degree to which the resources matched the participant’s needs (10 representing best match) was 7.42 (*SD* = 2.0), and the average 1–10 rating of the degree to which the resources were found to be helpful (10 representing most helpful) was 6.96 (*SD* = 2.35).

#### Less-Privileged Subgroup Analysis of Acceptability of The Postpartum Toolkit

Subgroup (*n* = 51) acceptability ratings were found to be slightly lower than the overall sample, with an average rating of helpfulness of 6.9 (*SD* = 2.46) and match to needs of 7.39 (*SD* = 2.1).

### 3.4. Pilot Efficacy of The Postpartum Toolkit

Table 3 shows the results of the pilot-efficacy analyses. Prior to analyzing differences in mean BIMF score from baseline to follow-up (primary outcomes of interest), a BIMF change score variable was computed and tested for normality. BIMF change score was found to be normally distributed, as assessed by a Shapiro–Wilk’s test (*p* = 0.258) as well as by visual inspection of a histogram and normal Q-Q plot of the BIMF change score. A matched pairs *t*-test was then performed, and results indicated that participant mean score on the BIMF was significantly higher at follow-up (*M* = 92.38, *SD* = 12.55), compared to baseline (*M* = 87.88, *SD* = 12.28); *t* (123) = −5.07, *p* < 0.001, and *d* = 0.46, indicating an improvement in functioning. To examine if there were particular functioning items that were driving this change, item level averages for BIMF score at baseline and follow-up were calculated (see Appendix F). These descriptive findings do not seem to highlight any particular item as seeming to drive the change, though scores on item 6 (“There are people in my life that I can trust to care for my baby when I need a break”) increased the most from baseline to follow-up.

Prior to analyzing differences in mean PSS-10, HADS-A, and HADS-D score from baseline to follow-up (secondary outcomes of interest), a change score variable was computed for each measure and that change variable was tested for normality. All change scores were determined to be normal by visual inspection of a histogram and normal Q-Q plot. Matched pairs *t*-tests were then performed for all four outcome variables. Results indicated that participant mean score on the HADS-A was significantly lower at follow-up (*M* = 7.12, *SD* = 3.73) compared to baseline (*M* = 7.99, *SD* = 4.16); *t*(123) = 3.18, *p* = 0.002, and *d* = 0.29. For the HADS-D, results revealed that participant mean score on the HADS-D was significantly lower at follow-up (*M* = 4.6, *SD* = 3.24) compared to baseline (*M* = 5.44, *SD* = 3.33); *t*(123) = 3.6, *p* < 0.001, and *d* = 0.32. Results for the PSS-10 revealed that participant mean score on perceived stress was significantly lower at follow-up (*M* = 16.26, *SD*= 5.96) compared to baseline (*M* = 18.16, *SD* = 6.29); *t*(123) = 4.07, *p* < 0.001, and *d* = 0.37.

#### Additional Exploratory Efficacy Analysis

In order to provide additional information about The Postpartum Toolkit’s effectiveness, a mixed ANOVA was conducted that examined change in outcome variables (BIMF, PSS-10, HADS anxiety, HADS depression) for individuals who reported use of the tools vs. those who reported they had not used the tools. Table 4 displays descriptive statistics of outcome measures.

For the BIMF, there was a significant main effect of time (i.e., a significant improvement in score from baseline to follow-up): *F* (1,122) = 23.38, *p* < 0.001, and ηp^2^ = 0.161, but there was not a significant main effect of condition (i.e., whether participants reported using the tools): *F* (1,122) = 0.266, *p* = 0.607, and ηp^2^ = 0.002. There also was not a significant time by condition interaction effect: *F* (1,122) = 3.42, *p* = 0.067, and ηp^2^ = 0.027.

For the PSS-10, there was a significant main effect of time (i.e., a significant improvement in score from baseline to follow-up): *F* (1,122) = 14.58, *p* < 0.001, and ηp^2^ = 0.107, but there was not a significant main effect of condition (i.e., whether participants reported using the tools): *F* (1,122) = 0.10, *p* = 0.754, and ηp^2^ = 0.001. However there was a significant time by condition interaction effect: *F* (1,122) = 4.6, *p* = 0.034, and ηp^2^ = 0.036. Comparisons using paired sample *t*-tests revealed that participants who endorsed using the resources in The Postpartum Toolkit had significantly lower stress scores at follow-up compared to baseline: *t* (69) = 4.99, *p* < 0.001, and *d* = 0.60. However, for individuals who did not report using the resources, there was no significant difference between stress score at baseline and stress score at follow-up: *t* (53) = 1.00, *p* = 0.320, and *d* = 0.14.

For the HADS-Anxiety, there was a significant main effect of time (i.e., a significant improvement in score from baseline to follow-up): *F* (1,122) = 9.09, *p* = 0.003, and ηp^2^ = 0.069, but there was not a significant main effect of condition (i.e., whether participants reported using the tools): *F* (1,122) = 1.37, *p* = 0.244, and ηp^2^ = 0.011. There also was not a significant time by condition interaction effect: *F* (1,122) = 1.15, *p* = 0.286, and ηp^2^ = 0.009.

For the HADS-Depression, there was a significant main effect of time (i.e., a significant improvement in score from baseline to follow-up): *F* (1,122) = 11.24, *p* = 0.001, and ηp^2^ = 0.084, but there was not a significant main effect of condition (i.e., whether participants reported using the tools): *F* (1,122) = 0.85, *p* = 0.358, and ηp^2^ = 0.007. However, there was a significant time by condition interaction effect: *F* (1,122) = 3.94, *p* = 0.049, and ηp^2^ = 0.031. Comparisons using paired sample *t*-tests revealed that participants who endorsed using the resources in The Postpartum Toolkit had significantly lower depression scores at follow-up compared to baseline: *t* (69) = 3.95, *p* < 0.001, and d = . 47. However, for individuals who did not report using the resources, there was no significant difference between depression score at baseline and depression score at follow-up: *t* (53) = 0.99, *p* = 0.35, and d = 0.13.

### 3.5. Participant Suggestions for Improving the Postpartum Toolkit

Participants had excellent suggestions with regard to ways in which the tool could be improved to increase engagement. Many of these suggestions centered on the format in which the tools were presented, as several participants reported that the text-based format was not found to be user-friendly and in fact, in several cases, was reported as “overwhelming.” Alternative formatting suggested by participants included the following: spreading out information (dividing the resources into “chunks”), using a podcast format, breaking up the text with more images or graphics, or delivering the information in the form of something like an Instagram post.

As part of their feasibility and acceptability questionnaire, participants were asked about additions they believe ought to be added to The Postpartum Toolkit Checklist (the measure used to determine which resources are presented to participants in their personalized feedback). Participants suggested adding items inquiring about the following: how far into the postpartum the individual is, baby health (e.g., chronic health diagnosis; NICU hospitalization), non-baby-related stressors (e.g., partners looking for jobs, COVID-19), “giving self grace”, preparation before baby arrives, and relationship with partner.

In addition, as part of their feasibility and acceptability questionnaire, participants were asked about resources they believed ought to be added to toolkit resources. Participant suggestions concerned the following: relationship functioning with romantic partners and other family members, COVID-19 (specifically, the negative impact of COVID-19 on employment), breastfeeding, local resources (midwives, counselors, lactation consultants), support groups, and tools for understanding a baby’s development.

Additionally, participants were asked in the feasibility and acceptability questionnaire about other resources they were using that were not included in their toolkit resources. Twenty-four participants (19.2% of the sample) endorsed using other resources. When asked to specify the resources used, participants cited the following: support groups, therapists, audiobooks, online resources, meditation, pelvic floor exercises, police/lawyer, parenting classes, pediatrician, and baby-milestone apps.

## 4. Discussion

In this study, data on the feasibility, acceptability, and pilot efficacy of an online tool based upon qualitative work conducted with the population of interest was collected.

Overall, The Postpartum Toolkit was found to be acceptable, with average ratings of both match with needs as well as usefulness (with 10 indicating a better match/higher degree of usefulness) reaching close to 7 out of 10, a metric used in comparable examinations of acceptability in other interventional work [50]. The majority (56%) of the 124 completers reported using at least one resource. As mentioned, insufficient time and forgetting about the resources were the most common barriers to use. On average, about 25% of the participants offered a resource reported that they used or intend to use that resource, though the resource with the highest percentage of use or intended use concerned self-care, reinforcing previous findings that self-care is an area of unmet need for the postpartum population [51]. This is further bolstered by the statistic that two of the checklist items with the lowest percentage of endorsed strengths related to self-care and involvement in activities outside of the home (see Table A2, Appendix E). Conversely, one of the least-used resources concerned infant-care education, which is noteworthy, as much prenatal education focuses on this topic. Perhaps these findings indicate that participants already had access to infant-care education, or it may be further evidence of a gap between patient and provider opinion on what training or information is most relevant in preparing for the postpartum transition. Additionally, despite prior evidence that self-guided interventions are efficacious [41], participants seemed to prefer having more interaction and guidance. Thus, in summary, the tools were deemed useful and a match to participant need, but participants reported barriers to utilization of the resources.

Self-report ratings of function, stress, and symptoms of depression and anxiety were statistically significantly different at follow-up compared to baseline. As mentioned, changes in all measures was in the direction that would indicate improved wellbeing (average score on function went up, average score on symptoms of stress, anxiety, and depression went down). For comparison, a Visiting Moms intervention (a preventative program providing social support to new birth parents) observed an average change of about 16 points on the BIMF over the course of about a year of visits [52]. The Visiting Moms study was conducted in a similar population to the current study (both were conducted in a sample endorsing minimal to mild depressive symptomatology), and the current study observed nearly a third of this change (average change of about five points on the BIMF) over the course of only six weeks. The Postpartum Toolkit also has the advantage of increased accessibility and flexibility (the parents can engage with the resources in their own time and the scheduling of additional appointments is not required). In addition, as described above, an additional exploratory analysis comparing change in outcome measures for individuals who reported use of resources provided by the tool compared to those who did not report using the resources provided by the tool was performed. Individuals who reported using the resources provided by the tool were found to have significantly lower stress and depression scores at follow-up compared to baseline. Individuals who did not report using the tool’s resources did not have significantly lower stress and depression scores at follow-up compared to baseline measurements. Though this analysis is confounded by the fact that the group who did not report using the tool’s resources is not a true control group (both groups received the intervention), it is an additional signal of the efficacy of The Postpartum Toolkit.

With regard to participant suggestions for improving The Postpartum Toolkit, it is interesting to note that several of these suggestions for additional resources (local resources, breastfeeding resources, statements normalizing the transition to parenthood, support groups, information about baby’s development) were included in the toolkit resources (see Appendix B). It is possible the individuals suggesting these additions were not presented with these resources in their feedback (suggesting that our mapping of resources could perhaps be improved), or perhaps barriers to engagement and issues with user-friendliness led participants to miss the presentation of these resources. It is also possible the users were providing these suggestions with others in mind, though they themselves did not endorse that they would like support with these things (and, thus, they were not presented with these resources and were unaware that they were already included in the tool).

### Limitations and Future Directions

There are several limitations to this project that bear mentioning. First, while these findings are promising, there are several considerations to take into account when interpreting these results. First, effect sizes for the analyses were small to moderate, and change in average score from baseline to follow-up was less than one point on both the HADS-A and the HADS-D. Thus, the clinical significance of the change is rather small. In addition, while the average level of perceived stress decreased from baseline to follow-up, average score of stress in the sample remained high at follow-up compared to the general population (the average PSS-10 score at follow-up was 16.5, and population norm is 13.7). Additionally, as mentioned, given that over half the sample was in the first six months postpartum when they enrolled in the study, this change may be explained by the natural history of postpartum adjustment, which remains largely unstudied. An efficacy trial using a randomized controlled trial design to better control for factors such as the natural history of postpartum adjustment would provide more convincing evidence of the efficacy of the tool to impact postpartum functioning and emotional wellbeing. If efficacy was proven following testing in a randomized controlled trial, the tool could be made available to postpartum birth parents, perhaps through hosting it on a website or through a mobile health application.

Additionally, it is important to acknowledge the larger cohort effect of the COVID-19 pandemic, as these data were collected between February and June 2021. Thus, there are other factors related to the pandemic that may have been impacting scores on outcome measures of emotional distress, such as adjustment to the restrictions of quarantine, or the lifting of restrictions.

Third, our sample was rather homogenous, and, in general, of a more privileged background. Thus, it would be important for future work to seek feedback and pilot data on this tool from a more diverse sample of birth parents. Given restrictions in place to manage the spread of the COVID-19 pandemic, recruitment for the study took place almost entirely online. General population-level research suggests that this recruitment venue may have influenced the sample, as social media users are more likely to possess a higher income and levels of education [53]. Additionally, the first author’s prior experience with recruiting for a qualitative project in the population of postpartum mothers found that using both in-person and online methods seemed to inadvertently target different demographic groups (those who were recruited through a remote means were majority White and reported higher estimated household income) [14]. A subgroup analysis of the less-privileged subgroup of participants was performed to examine whether baseline characteristics, feasibility, and acceptability differed. Overall, the sample and the sub-groups were statistically indistinguishable from one another. However, it will be especially beneficial for future work to seek out the perspective of a more diverse population, including transgender birth parents, given the dearth of research in this population. The importance of studying transgender birth parents was underlined by the finding that both parents who identified as male reported spending time in the NICU. Though the sample size was extremely small, this finding is noteworthy given the current dearth of research on birth outcomes for transgender men and previous findings that institutional erasure functions as a barrier to receiving routine perinatal care [3]. This leads one to wonder whether barriers to care might have impacted these participants’ pregnancy and birth experiences, ultimately leading to their infants’ NICU hospitalizations.

In addition to recruiting a more diverse sample, it would also be important for future work to involve integrating participant feedback into the tool to improve feasibility of use. Given feedback suggesting that changing resource formatting could increase user friendliness, it would be particularly important to consider altering the text-based format in order to improve resource engagement with a future iteration of the tool.

## 5. Conclusions

In conclusion, postpartum functioning is of critical importance to birth parents and their infants throughout the postpartum year. However, despite recognition of the importance of the postpartum year, birth parents face many barriers in accessing healthcare and relevant support resources. To address this, the feasibility, acceptability, and pilot efficacy were tested of an online tool based upon qualitative work with postpartum birth parents that provided parents with personalized, tailored resources to bolster functioning. The Postpartum Toolkit was found to be acceptable, but participants reported barriers to use, and made suggestions for improvement, including altering the text-based format to increase user-friendliness. Additionally, pilot-efficacy data suggests that this tool may be effective in improving postpartum functioning and emotional wellbeing. Therefore, a future iteration of the tool that incorporates participant feedback from a diverse population of birth parents could prove an effective means of delivering helpful support to an at-risk population, particularly in the era of a global pandemic, when in-person engagement is limited and the need for mental-health support is heightened.

## Figures and Tables

**Table 1:** Participant sociodemographics, reproductive history, and psychiatric history.

	Total (*n* = 125)
**Race**	*n* (%)
Asian	9 (7.2)
Black or African-American	3 (2.4)
Multiracial	3 (2.4)
Other	3 (2.4)
Prefer not to report	5 (4.0)
White	102 (81.6)
**Ethnicity**	
Hispanic or Latinx	10 (8)
Non-Hispanic or Latinx	115 (92)
**Gender**	
Female	123 (98.4)
Male	2 (1.6)
**Marital Status**	
Married	117 (93.6)
Partnered but not married	7 (5.6)
Single	1 (0.8)
**Access to support with infant care**	
No	10 (8)
Yes	115 (92)
**Religious Identity**	
Buddhist	3 (2.4)
Christian	71 (56.8)
Jewish	10 (8)
Not religious	36 (28.8)
Other	5 (4)
**Education**	
College graduate	47 (37.6)
Graduate degree	70 (56)
Did not complete high school	1 (0.8)
High school graduate/GED	4 (3.2)
Other	3 (2.4)
**Employment**	
Employed	102 (81.6)
Homemaker/stay at home	16 (12.8)
Other	7 (5.6)
**Income**	
Less than USD $25,000	3 (2.4)
USD $25,000–USD $74,999	23 (18.4)
USD $75,000–USD $124,999	37 (29.6)
USD $125,000–USD $174,999	22 (17.6)
More than USD $175,000	40 (32)
**Participant age (years)**	
18–25	5 (4)
25–35	85 (68)
35–45	34 (27.2)
46 or older	1 (0.8)
**Age of youngest infant at baseline**	
0–3 months	32 (25.6)
3–6 months	42 (33.6)
6–9 months	28 (22.4)
9–12 months	23 (18.4)
**Mode of delivery**	
Caesarean section	36 (28.8)
Vaginal	89 (71.2)
**Whether mode of delivery proceeded as planned**	
Yes	111 (88.8)
No	14 (11.2)
**Youngest infant singleton or multiple**	
Singleton	123 (98.4)
Multiple	2 (1.6)
**Number of children**	
1	67 (53.6)
2	49 (39.2)
3	6 (4.8)
4	2 (1.6)
More than 4	1 (0.8)
**Whether pregnancy of youngest infant was planned**	
Yes	108 (86.4)
No	17 (13.6)
**Infant born at term**	
At term	119 (95.2)
Preterm	6 (4.8)
**Whether youngest infant was hospitalized in a Neonatal Intensive Care Unit**	
No	109 (87.2)
Yes	16 (12.8)
**Whether participant experienced postpartum re-hospitalization**	
No	119 (95.2)
Yes	6 (4.8)
**Whether participant received treatment for infertility to conceive youngest infant**	
No	109 (87.2)
Yes	16 (12.8)
**Participant history of stillbirth or miscarriage**	
History of stillbirth or miscarriage	34 (27.2)
No history	91 (72.8)
**Participant psychiatric history**	
Negative history of psychiatric or psychological condition	87 (69.9)
Positive history of psychiatric or psychological condition	38 (30.4)
**Experienced a medical condition complicating pregnancy**	
No	74 (59.2)
Yes	51 (40.8)
**Medical conditions experienced during pregnancy**	
Asthma in pregnancy	12 (9.6)
Diabetes related	12 (9.6)
High blood pressure during pregnancy	18 (14.4)
Obesity in pregnancy	14 (11.2)
Other medical condition ^a^	12 (9.6)

^a^ Reported medical conditions included: cerebral spinal fluid leak, cholestasis, hyperemesis, hypothyroidism, intrahepatic cholestasis of pregnancy, kidney disease, pelvic girdle pain, preeclampsia, preterm labor at 32 weeks, rheumatoid arthritis, Hashimoto’s thyroiditis, suspected cavernous hermangioma of the uterus, Von Willebrand disease, and mild hemophilia.

**Table 2:** Participant report of feasibility and acceptability.

		Total (*n* = 124) ^a^
**Did you use any of the resources?**		*n* (%)
Yes		70 (56.5)
No		54 (43.5)
**How often did you refer to/look at the feedback that was sent to you?**		
A few times		56 (45.2)
I only looked at it once		54 (43.5)
Often		2 (1.6)
Several times		12 (9.7)
**If you did not use these resources, what got in the way? ^b^**		
Didn’t find the resources appealing		17 (13.7)
Didn’t have time		50 (40.32)
Forgot about it		42 (33.87)
Other		17 (13.7)
COVID-19 made use difficult		3 (2.42)
Cost		4 (3.23)
Resources not useful		6 (4.8)
Formatting made difficult to access		6 (4.8)
**Would you have been more likely to use these resources if you had a person introducing them to you rather than looking into things yourself?**		
Yes		72 (58.1)
No		52 (41.9)
	***M ± SD***	**Range**
**On a scale from 1–10 how much did you feel the feedback matched your needs (with 10 being the best match)?**	7.42 ± 2.0	1–10
**On a scale from 1–10 how helpful did you find your personalized resources (with 10 being the most helpful)?** ^c^	6.96 ± 2.35	1–10

^a^ A total of 125 participants completed the study, 1 of which was not presented with resources as they did not endorse any weaknesses in The Postpartum Toolkit Checklist and thus were not presented with resources. ^b^ Participants could select multiple options. ^c^ Participants were asked for a rating of helpfulness at both the mid-study timepoint (three weeks after baseline) and at the final study timepoint (six weeks after baseline). The results seen here are ratings of helpfulness from the final six-week timepoint.

**Table 3:** Results of pre/post analysis for quantitative self-report measures.

Measure	Baseline Score		Follow-Up Score		*t* (123)	Two-Sided *p*	Cohen’s *d*
	*M*	*SD*	*M*	*SD*			
Barkin Index of Maternal Functioning (BIMF) ^a^	87.88	12.28	92.38	12.55	−5.07	<0.001	0.46
Perceived Stress Scale-10 (PSS-10) ^c^	18.16	6.29	16.26	5.96	4.07	<0.001	0.37
Hospital anxiety and Depression Scale-Anxiety Subscale (HADS-A) ^b^	7.99	4.16	7.12	3.73	3.18	0.002	0.29
Hospital Anxiety and Depression Scale-Depression Subscale (HADS-D) ^b^	5.44	3.33	4.6	3.24	3.6	<0.001	0.32

^a^ Scores to the right of 80 on a number line indicate ideal functioning. ^b^ Scores of 8 or higher on either subscale indicate clinically elevated symptoms. ^c^ The population norm is a score of 13.7.

**Table 4:** Descriptive statistics for outcome measures by resource-use condition (used vs. not).

	Used Resources (*n* = 70)	Did Not Use Resources (*n* = 54)
BIMF Baseline *M ± SD*	86.7 ± 12.61	89.41 ± 11.78
BIMF Follow-Up *M ± SD*	92.63 ± 12.55	92.06 ± 12.66
PSS-10 Baseline *M ± SD*	18.46 ± 6.8	17.78 ± 5.61
PSS-10 Follow-Up *M ± SD*	15.69 ± 5.84	17.00 ± 6.09
HADS Anxiety Baseline *M ± SD*	8.46 ± 4.59	7.39 ± 3.48
HADS Anxiety Follow-Up *M ± SD*	7.33 ± 3.85	6.85 ± 3.59
HADS Depression Baseline *M ± SD*	5.41 ± 3.45	5.46 ± 3.20
HADS Depression Follow-Up *M ± SD*	4.19 ± 3.01	5.15 ± 3.46

BIMF = Barkin Index of Maternal Functioning; PSS-10 = Perceived Stress Scale-10; HADS = Hospital Anxiety and Depression Scale.

## Data Availability

The data presented in this study are available on request from the corresponding author.

## Appendix A. The Postpartum Toolkit Checklist

Listed below are things that parents told us help them to manage all the tasks and responsibilities that come along with having a new baby. For each one of these things, select which answer fits best for you. Everyone has different strengths and challenges, so we will use your responses to give feedback that is personalized to you.

1. When things don’t go well, I don’t blame myself.	This is not an issue for me.	This is going ok for me, but could be better.	I’d like help with this.
2. When trying new things, I do all of the following: I am patient with myself and don’t expect myself to get things right away I feel comfortable pushing myself beyond my comfort zone I can be flexible with my plans and expectations.	**This is a strength for me.**	**This is going ok for me, but could be better.**	**I’d like help with this.**
3. My baby and I are bonding.	**This is going well for me.**	**This is going ok for me, but could be better.**	**I’d like help with this.**
4. Most of the time my baby’s crying doesn’t overwhelm me.	**This is a strength for me.**	**This is going ok for me, but could be better.**	**I’d like help with this.**
5. Most of the time I am able to calm myself down when I have strong emotions (such as stress, worries, anger) or distressing thoughts.	**This is a strength for me.**	**This is going ok for me, but could be better.**	**I’d like help with this.**
6. I have people in my life that help to make sure I’m taking care of myself. For example, people in my life check-in with me about taking care of myself, or they help me to take care of myself by doing things like watching the baby.	**This is going well for me.**	**This is going ok for me, but could be better.**	**I’d like help with this.**
7. I am able to get the materials I need to take care of the baby and myself (such as diapers and food).	**This is not an issue for me.**	**This is going ok for me, but could be better.**	**I’d like help with this.**
8. I am getting more experienced with taking care of my baby.	**This is going well for me.**	**This is going ok for me, but could be better.**	**I’d like help with this.**
9. When I do things well, I am able to give myself credit for it.	**This is a strength for me.**	**This is going ok for me, but could be better.**	**I’d like help with this.**
10. *If you have people in your life who help and support you*: when I am talking to people in my life who help or support me, I do all of the following: I can be open and honest I can trust people to help me I can ask for help and take help when it is offered	**This is a strength for me.**	**This is going ok for me, but could be better.**	**I’d like help with this.**
11. I have been able to get my baby into a routine.	**This is going well for me.**	**This is going ok for me, but could be better.**	**I’d like help with this.**
12. When I have a question about parenting or early motherhood, I know how to get an answer that I trust.	**This is going well for me.**	**This is going ok for me, but could be better.**	**I’d like help with this.**
13. I am involved in activities outside of the house (such as friendships, hobbies, professional or volunteer work).	**This is going well for me.**	**This is going ok for me, but could be better.**	**I’d like help with this.**
14. I know myself (when I need to ask for help, what I need, what makes me feel better when I feel down, worried, or stressed).	**This is a strength for me.**	**This is going ok for me, but could be better.**	**I’d like help with this.**
15. My home environment is safe and stable.	**This is not an issue for me.**	**This is going ok for me, but could be better.**	**I’d like help with this.**
16. I know that taking care of myself is important, and guilt does not get in the way of taking care of my own needs.	**This is a strength for me.**	**This is going ok for me, but could be better.**	**I’d like help with this.**
17. I am able to sleep at night when my baby is sleeping.	**This is going well for me.**	**This is going ok for me, but could be better.**	**I’d like help with this.**
18. When thinking about social pressures, I don’t do any of the following: feel like I have to reach the expectations of others worry about other people’s judgments of me compare myself to others.	**This is a strength for me.**	**This is going ok for me, but could be better.**	**I’d like help with this.**
19. I plan and manage time well.	**This is a strength for me.**	**This is going ok for me, but could be better.**	**I’d like help with this.**
20. I have people in my life that I can talk to openly and honestly about my feelings and experiences.	**This is not an issue for me.**	**This is going ok for me, but could be better.**	**I’d like help with this.**
21. I have people to help me with childcare and household management (cooking, cleaning, shopping, etc.).	**This is not an issue for me.**	**This is going ok for me, but could be better.**	**I’d like help with this.**
22. I understand what my baby is trying to tell me (through their cries, body language, etc.).	**This is a strength for me.**	**This is going ok for me, but could be better.**	**I’d like help with this.**
23. *If you or your co-parent are currently working*: my workplace/my partner’s workplace has been understanding and flexible since I have had my baby.	**This is not an issue for me.**	**This is going ok for me, but could be better.**	**I’d like help with this.**
24. I am not experiencing physical pain or discomfort.	**This is not an issue for me.**	**This is going ok for me, but could be better.**	**I’d like help with this.**

## Appendix B. Alphabetized List of Resources (as Presented to Participants with the Exception of the Removal of Embedded Videos; There is Text to Indicate where the Videos Would Appear)

[*the following language appeared before the curated list of resources*] Below you will find information and suggestions that are have been selected specifically for you. You will notice that, above each suggestion, it will say in gray letters what you answered in the checklist that lead to that particular suggestion being selected (or, in other words, why we think that suggestion might be helpful for you). The selection of helpful resources was created using suggestions from parents like you who have recently had a baby. You may notice that we incorporated real words from parents into these materials. In addition to parents, we also worked with health care experts to put this “toolkit” of information and suggestions together. If you would like information about the research backing up these recommendations, you can contact a study team member at ama482@drexel.edu.

While we hope that you find this information helpful, it should be noted that these resources **do not replace** meeting with a healthcare professional.

A	**Question self-blame** Parents have a tendency to blame themselves, even when they know things are outside of their control. As one mom said, “*Everything that happens is something that you could have affected. And so if things are going poorly you must be doing poorly. To some extent, it’s like some of us get lucky to have things go well at certain points in time and some of us don’t, and that affects how well we can feel like things are getting better*.” When you feel yourself taking on blame for something that has gone wrong, ask yourself if your view is accurate. Was what happened really something you could have controlled? This takes practice, but there are exercises you can perform which can get you into the habit of questioning the blame you may be putting on yourself. One exercise is called “Retelling your own story” from *Getting Our of Your Mind and Into Your Life* by Dr. Steven Hayes. This exercise involves writing down a paragraph to describe a recent event that brought about thoughts of self-blame. After you finish, underline the facts of the story (the aspects of the story that everyone would agree are true regardless of their point of view). Then, write a different story including the same facts. This is a great way of showing you that facts become relevant because of the story that you tell around them, and you have more control over this story than you may think. Here is another exercise: in a paper journal or a notepad app in your phone, write down the “evidence” that you are to blame for a recent event that brought about thoughts of self-blame. Then, write down the “evidence” against blaming yourself. Remember, you want to use *fact*, not guesses, interpretations, or opinions. This may be hard to do at first, but over time you will become better at seeing the evidence from both sides, and you will see that your responses are not driven by events alone, but also your thoughts and emotions in response to events.
B.	**Increase self-compassion** Work to foster *self-compassion*, which helps you to see yourself (both strengths and weaknesses) without judgment, and to accept yourself as you are. In short, it is learning to treat yourself like a good friend. As one mother told us: “*I think it’s important to not put in too much from yourself. So give yourself some grace and knowing by yourself that you’re doing a good job, even if you don’t feel it at the time*.” There is research to show that when we are more self-compassionate, and less self-critical, we are better off. Self-compassion is linked to positive emotions and better overall outcomes, while self-criticism is linked to negative emotions and worse overall outcomes. While we might think being more critical of ourselves will make us more successful, that is not what the research on self-compassion suggests. To give yourself a taste of what this feels like, try the following few steps taught by self-compassion expert Dr. Kristin Neff when you are feeling low, sad, or stressed: Validate your own feelings. Tell yourself “this is hard right now.” Acknowledge that feeling badly, and even suffering, is part of the human experience. You are not alone in this. Bring some words of kindness to yourself. You might say to yourself the things you would say to a good friend such as “I’m sorry this is hard, what can I do to help? I am here for you.” If you want to learn more, there is a free course about self-compassion available at the following link: https://product.soundstrue.com/power-of-self-compassion/free-video-series/?ck_subscriber_id=597375296 The University of California at Los Angeles also has a free meditation that works to foster a sense of loving kindness, give it a try at the following link: https://www.uclahealth.org/marc/mpeg/05_Loving_Kindness_Meditation.mp3
C	**Consider seeking professional mental health support** It can be helpful to have the support of a psychotherapist or counselor to sort through everything that you are experiencing. The transition to parenting a new baby is tough, and a psychotherapist or counselor can help to support you through all the changes (in your body, relationships, identity) that are happening, and teach you tools to help manage stressors you may be experiencing. Also, as someone outside of your circle of family and circle of friends, you can feel comfortable speaking openly about your relationships. You don’t have to fear that you are putting your thoughts and emotions onto someone else, because it is a psychotherapist or counselor’s job to listen and support you. As one mother described what helped her to adjust after having a new baby: “ *I’m going to therapy. I’m talking, I’m communicating, I’m asking for help, I’m asking for support, and I’m getting past day by day*.” Here are some places to start when trying to find a psychotherapist or counselor: Postpartum Support International (https://www.postpartum.net/get-help/help-for-moms/) is a good place to look for help in your area because they have information about psychotherapists, counselors, and programs that specifically work with people who have recently had a baby. You can call or text their toll-free helpline at 1-800-944-4773(4PPD) for basic information, support, and resources in your area. If you have health insurance, you can call your insurance company to ask for psychotherapists or counselors in your area. Go to the website Psychology Today (https://www.psychologytoday.com/us) and use their “find a therapist” search bar. You can enter your zip code to find providers near you. Once you have entered your zip code and press the enter key, results will appear. A column will also appear on the left-hand side of the screen and will allow you to select even more specific information about the psychotherapist or counselor you are looking for (your insurance, the issues you would like to focus on, etc.). Here is a specific directory you can use to look for psychotherapists with expertise treating Black women: https://providers.therapyforblackgirls.com/ If you do not have health insurance, you can consider searching local training clinics that offer a sliding fee scale they do not have one set price but set a price based upon how much you can pay. To do this, search on the internet “psychology training clinics near me”. Or, you can try to find a Volunteers in Medicine clinic near you and see if they offer mental health services: https://vimamerica.org/clinics/ There are also several websites organized by professional associations that have searchable directories of mental health providers: The Substance Abuse and Mental Health Services Administration: https://findtreatment.samhsa.gov/ The American Psychological Association: https://locator.apa.org/ The Association for Behavioral and Cognitive Therapies: https://www.findcbt.org/FAT/ Some people benefit from an intensive outpatient program (IOP), which usually involves receiving care from multiple providers and attending more frequent appointments (usually multiple appointments a week) as part of one program. If you are feeling that it is hard to get through the day due to low mood, lots of worries, or other concerns related to your mental health, one of these programs may be helpful for you. See the list below for the locations of intensive programs in the United States: https://www.postpartum.net/get-help/intensive-perinatal-psych-treatment-in-the-us/
D	**Use positive affirmations** Use positive affirmations when you are feeling anxious and distressed about trying something new. Research shows that this can help to increase your self-worth and improve your mindset. Positive affirmations are positive phrases or statements that you can use to encourage yourself. You can post them around your house or car or you can simply repeat them. Some examples are below. If you don’t connect with any of these, you are welcome to write your own! I am allowing myself space to grow and learn I am letting myself just “be”, without judgment I am accepting my emotions and letting them serve their purpose For more information, visit the following link: https://theblissfulmind.com/positive-affirmations-list/
E	**Get some distance from your thoughts** Sometimes you may have thoughts that feel much bigger than just thoughts. For example, you may have a thought that you are not meeting your expectations, you may think that you should feel guilty for taking time for yourself, or you may have a thought that others are judging you to be a bad parent. As one mom told us: “*Expectations get in your way a lot because you think it’s going to be one way and then…it can feel like a letdown. You can feel like you are failing one after the other, when really it’s just because your expectation is not being met, which is not the same as failing. Bottle-feeding your baby is not the same as failing at breastfeeding. And it probably took me until a month ago to realize*.” When you think these things, you have an emotional response, and it may change the way you see yourself. This can be distressing. While these thoughts will be a part of life, you can work to gain a little bit of distance from the thoughts, which will in turn lessen their effect on our emotions and understanding of yourself. There are several techniques for making your distressing thoughts less powerful. Listed below are a few discussed by Dr. Steven Hayes, You can “thank your brain” for having the thoughts. You even just say to yourself “I notice myself having the thought that…” Or you can engage in a meditation, taking a few moments to visualize your thoughts like leaves on a stream. Place each thought that enters your mind on a leaf on the stream and letting it float past. For guidance through this exercise check out the video below, created by Inner Melbourne Clinical Psychology. It is also available at this link: https://www.youtube.com/watch?v=00AbNXNLUUs While these may seem silly, doing things like this will remind you that your thoughts are just thoughts, and allowing yourself to observe them rather than have them “take over” can have a powerful influence on the distress you feel when these thoughts arise. [EMBEDDED LEAVES ON A STREAM VIDEO]
F	**Learn about bonding with your baby** Remind yourself that there is no one “right” way to feel about your relationship with your baby, even if others expect you to feel a certain way. Give yourself time to get to know your baby and trust that you will become closer to the baby over time. In the meantime, the tips below might help you to find new, fun ways to grow closer to your baby. Use the links below to learn about how your baby is developing, and what you can do to encourage growth in your baby while also bonding with them. https://talkingisteaching.org/resources https://www.zerotothree.org/resources/161-development-from-birth-to-12-months-old-forming-a-trusting-bond-to-nurture-learning#downloads https://www.babycenter.com/2_creating-an-attachment-with-your-baby_10350318.bc You may also want to try the free app Vroom, which offers a set of tools and resources to turn everyday moments into “brain building” moments that you and your infant can share together. They also have documents that you can print if you would prefer to not use the app! Learn more at this link: https://www.vroom.org/ You may also like the apps BabySparks (https://babysparks.com/) or Kinedu (https://www.kinedu.com/) help parents support healthy development through play. Use the links below to learn about activities that experts suggest can be helpful for bonding with your baby: https://www.zerotothree.org/resources/1077-activities-for-bonding-and-learning-from-birth-to-12-months https://www.zerotothree.org/resources/2924-6-ways-to-bond-while-you-re-on-the-move The video below was created by YMC Motherhood Unfiltered and is also available at the following link: https://www.youtube.com/watch?v=4VuEIeDrwAM [EMBEDDED YMC MOTHERHOOD UNFILTERED VIDEO] If you have the time, build infant massage into your daily routine. Evidence shows it has lots of benefits for you and baby, including helping you to bond with one another. The video below, created by Cincinnati Children’s, shows you how to get started and is also available at this link: https://www.youtube.com/watch?v=CJPu_bDus3Q [EMBEDDED INFANT MASSAGE VIDEO] Try to be present with your baby. As best you can, avoid activities like scrolling on your phone during time with your baby. Also, keep in mind that these opportunities for bonding may not always be at the times you planned. When your baby is in a playful mood, take advantage of this time. Lean in and let yourself enjoy it.
G	**Calm your baby and yourself through crying spells** Infant crying is very distressing for parents. One mom described how upsetting crying can be by saying: *“[My baby] cried for like 45 min straight and I mean it’s so wearing…It’s the worst thing*.” It can be helpful to learn from experts about infant crying and how to soothe your baby so that you feel prepared to calm your baby the next time they cry. Helpful links are listed below: https://www.zerotothree.org/resources/272-coping-with-crying-in-babies-and-toddlers The video below was created by Eugene Pediatrics Associates and is also available at this link: https://www.youtube.com/watch?v=Y6TVv85NPlw [EMBEDDED VIDEO] The video below, created by Real Happy Endings, talks about the Book Happiest Baby on the Block by Dr. Harvey Karp. It gives information about calming very young babies (first few months of life). It is also available at this link: https://www.youtube.com/watch?v=crdQy8zliZw. [EMBEDDED VIDEO] If you suspect that your baby is experiencing discomfort, some ways to relieve this are: **sucking** (either to feed or on a pacifier), **security** (swaddling the baby, being wrapped up reminds them of being in their mother’s belly which calms them down), **shushing** (making a shhhh sound or playing white noise) **One note, if your baby is in the hospital or experiencing a medical condition, check with their doctor first before trying new things. ** The video below, created by FirstCry Parenting, is about crying at night. It is also available at this link: https://www.youtube.com/watch?v=3ckmDR7oNbQ [EMBEDDED VIDEO] However, even when you have information about why babies cry and how to soothe them, sometimes you still won’t be able to stop them from crying. If it is safe to do so *(you can leave baby in a safe place or can have someone else take over care)* during those times, allow yourself to take a break and do something calming for yourself. You might consider taking “shifts” with a co-parent, friend, or other support person. Allowing yourself some time away from the baby can reduce your stress and help you feel more able to tackle the problem of soothing your infant. Also, your baby can sense your energy, if you are calmer, it is likely this will help your baby to become calmer. One way to calm yourself in the moment is through your breathing. Take breaths in through your nose and out through your mouth. Try your best to make the outbreath last longer than the inbreath, as the outbreath has a strong impact on slowing down your heart rate and calming your body down. Also make sure you are “belly breathing” (your belly rises with your inhale; you might imagine wearing pants that are too big and breathing out to hold the pants up). Belly breathing prevents you from shallow “chest breathing” which can increase your anxiety. The video below, created by The Somerville Foundation, shows you how to breathe in a calming way. It is also available at this link: https://www.youtube.com/watch?v=4e3Csho8CBw [EMBEDDED VIDEO] For other calming breathing techniques check out this article: https://greatist.com/happiness/breathing-exercises-relax#Why-controlled-breathing?
H	**Gain some tools to help you manage strong emotions** Especially with all of the changes you are going through (in your body, in your relationships, to your sleeping routine), it is normal to have strong emotions like frustration, stress, or anxiety. One way to calm yourself when you are feeling strong emotions is to use mindfulness. Mindfulness is a technique that teaches you to focus on the present moment, acknowledging the thoughts, emotions, and sensations you are experiencing, without judgment. Mindfulness has been shown to decrease sadness and stress and increase happiness and focus. As one mother described using mindfulness: “*In preparation for parenthood, I got into mindfulness and meditation, and so that really helped me sort of find space and calmness and ways to just breathe through any stressors or tension, and sort of reassure myself that all the moments are manageable*.” But, like any skill, it takes practice to “build up the mindfulness muscle” so that we can put it into play during distressing moments in life when we need it most. The good news is that there are a number of free online resources that you can use to learn about and practice mindfulness. We have collected some here: Mindfulness Coach Smartphone Application, free self-guided training in Mindfulness https://mobile.va.gov/app/mindfulness-coach Headspace Smartphone Application https://www.headspace.com/headspace-meditation-app UCLA Mindfulness meditations https://www.uclahealth.org/marc/mindful-meditations When you feel very strong emotions, like anger or feeling like you can’t stop thinking about and focusing on your worries (this is called “rumination”), there are also several techniques, discussed by Dr. Marsha Linehan, which can help you to calm yourself down and get through difficult moments. Self-soothing Use your five senses to focus on and savor something that is enjoyable to you. See below for some examples for each sense: **Vision**: look at the sunset, sunrise, or night sky **Hearing**: listen to music or a podcast that you like **Smell**: light a scented candle, smell a kitchen item like coffee beans or vanilla extract, or scented soap (like lavender) **Taste**: drink your favorite soothing drink like tea or hot chocolate **Touch**: take a hot shower or bath Improve the current moment. If distracting or self-soothing is not working, try to find the meaning in the current moment. If you are religious, pray. As one mom said, “ *I literally just pray, definitely pray…That calms me down. Even if it’s outside the kids, praying calms me down*.” Perform a body scan, an exercise in which you focus on your experience of sensations in your body. The video below, created by Vanderbilt University, walks you through how to perform a body scan. It is also available at this link: https://www.youtube.com/watch?v=Xo3PetVUcIc [EMBEDDED VIDEO] When other things don’t work, you can do certain things with your body that can help to calm your emotions. Here are some examples: Put cold water or ice packs to your face while holding your breath. This will initiate the human “dive reflex” which will reduce your emotional arousal very quickly. Do intense aerobic exercise (jumping jacks, jogging) of any kind for 20 min. This has been shown to decrease negative mood and give you a positive mood boost. **Be careful with this if you are still recovering from birth. You can consult your doctor about what exercise may be appropriate for you.** Use slow, paced breathing. The video below, created by The Somerville Foundation, shows you how to breathe in a calming way. It is also available at this link: https://www.youtube.com/watch?v=4e3Csho8CBw [EMBEDDED VIDEO] Use paired muscle relaxation, which involves relaxing your muscles while breathing out. The video below, created by Therapist Aid, shows you how to do a similar exercise called progressive muscle relaxation. It is also available at this link: https://www.youtube.com/watch?v=1nZEdqcGVzo [EMBEDDED VIDEO] If you are experiencing a lot of anxiety, there is a free application that you can download to your smartphone that will teach you research-backed techniques for taking active steps to manage your anxiety: https://www.anxietycanada.com/resources/mindshift-cbt/
I	**Consider a postpartum doula** Consider seeking support from a **postpartum doula**. A postpartum doula is a trained professional with knowledge of emotional and physical recovery from childbirth, infant soothing and newborn care, and mother-infant bonding. Having a strong support system is key to a healthy transition after having a baby, and a doula can play a major role in that support. You can think of a doula as someone who can “mother” you while you are adjusting to caring for your baby. For example, a doula can help to answer your questions and provide an extra pair of hands with infant care. For more information, check out this article: https://www.nytimes.com/2018/10/02/style/postpartum-doula.html If you are interested in finding a postpartum doula, here is some advice on how to start your search: Look online, the following websites have searchable directories of doulas, make sure you are searching for/selecting **postpartum** doulas: https://www.dona.org/what-is-a-doula/find-a-doula/ https://cappa.net/directory/ https://doulatrainingsinternational.com/find-a-doula/ A reproductive justice organization led by Black women, can help match Black parents with a doula https://www.roottrj.org/find-a-doula You can also ask about postpartum doulas from childbirth educators, or parenting support groups in your area. If you are concerned with the cost of a doula, here is some information about paying for postpartum doula services: Look for an organization that trains doulas in your community, you may be able to get a discount if a doula is newly trained. You may be able to pay for a doula from a flexible spending or health savings account. Many doulas operate on a sliding fee scale (they adjust their price based upon what you can pay). Look into Community Doula Programs, which pair women with doula support for low or no cost. Contact your local birth centers, hospitals, or state health department to find out whether there is a community doula program near you. For more information https://www.postpartum.net/get-help/birth-and-postpartum-doulas/
J	**Learn about the resources around you** Taking care of a baby is expensive. It can be stressful and upsetting to be in the struggle to afford baby supplies. However, below are some organizations that may help get you what you need. **Baby Supplies** Baby2Baby National Network Distributing Childcare Essentials: https://baby2baby.org/national-network/ Breast pump Contact your insurance provider and ask if they are able to provide you with a breast pump. Under the Affordable Care Act insurance plans **must** provide breastfeeding support, counseling, and equipment for the duration of breastfeeding Car seat Call your local United Way by dialing 211 on your phone Cribs The following organization provides education on the importance of practicing safe sleep for babies and provide portable cribs to families who, otherwise, cannot afford a safe place for their babies to sleep: https://cribsforkids.org/request-a-crib/ Diapers: The National Diaper Bank Network: https://nationaldiaperbanknetwork.org/get-help-now/ General Supplies Local churches and religious charities can provide support regardless of your personal faith. You can contact your local place of worship to learn about what is available in your area. Below are some religious programs and churches that offer support to low-income families. Catholic Charities USA: https://www.catholiccharitiesusa.org/find-help/ Jewish Federation of North America: https://www.jewishfederations.org/ Lutheran Services: https://www.lutheranservices.org/ United Methodist Church: https://www.umc.org/en/find-a-church Baby Box University Available in select states, provides free sleeping boxes (used like a crib) and samples of products like wipes and diapers. You can register for a free education program and get free baby supplies: https://www.babyboxco.com/classes/login **Food**: Women Infants and Children (WIC) Special Supplemental Nutrition Program: https://www.fns.usda.gov/wic Gives access to healthy food, nutrition education, breastfeeding guidance Caregivers with a low to medium income and those who are part of other programs such as foster care, medical assistance, or SNAP are eligible Feeding America, national network of food banks: http://www.feedingamerica.org/find-your-local-foodbank/ Supplemental Nutrition Assistance Program (SNAP): https://www.fns.usda.gov/snap/recipient/eligibility **Medical Care**: Find a Volunteers in Medicine affiliated clinic near you which provides free health care services to the uninsured and medically underserved: https://volunteersinmedicine.org/volunteers-in-medicine-clinic-directory/ **Free or reduced cost services** (food, medical care), search by zip code to see organizations near you: https://www.auntbertha.com/
K	**Learn about baby care** It takes time to gain experience in taking care of your baby. Each baby is unique. It is important to keep the learning curve in mind, and to be patient with yourself. However, there are also free and low cost educational materials available online that can help you feel more prepared for the challenges of taking care of a baby. These are organized below by topic. Keep in mind that you can also look for classes at hospitals, preschools, pediatrician offices, or social service facilities in your area **General infant care classes and trainings**: Low cost online parenting classes covering breastfeeding, sleep, infant CPR, baby care and more: https://www.tinyhood.com/online-parenting-classes Baby Box University has a number of free online classes on topics including breastfeeding, infant brain development, and newborn sleep behavior. You can also get rewards for completing the classes: https://www.babyboxco.com/classes/login **Breastfeeding** Stanford University short course on breastfeeding: https://www.classcentral.com/course/breastfeeding-10633 Lactation Link’s online breastfeeding course: https://lactationlink.com/free-6-day-course/ Women’s health.gov breastfeeding resources: https://www.womenshealth.gov/patient-materials/resource/guides Breastfeeding Class with Lactation Consultant: https://www.milkandlove.com.au/breastfeeding-online-classes-with-katie-james-ibclc/ The video below, created by The Yellow Nursery, has information on getting a good latch in breastfeeding. It is also available at this link: https://www.youtube.com/watch?v=OUqRVSeBpY8 [EMBEDDED VIDEO] **Sleep** Expert written resources on sleep: https://www.babysleep.com/ Baby Sleep Made Simple Program: https://www.youtube.com/playlist?list=PLTOm7KLBBnKph5OubZ9pO0EeHcw1jO9W- **CPR** Online CPR course from RedCross (approximately $35): https://www.redcross.org/take-a-class/classes/child-and-baby-first-aid%2Fcpr%2Faed-online/a6R0V0000015FV5.html
L	**Increase the power of positive emotions** The field of positive psychology, led by Dr. Martin Seligman, focuses on building positive qualities and involves techniques that increase how often and how deeply we experience positive emotions. One aspect of positive psychology that may be particularly helpful to you is increasing your awareness of your successes, and learning to appreciate them. This may be especially helpful because it can be hard to give yourself credit for all the good things you are doing as a parent. As one mom said “*It’s hard for people to give themselves positive feedback. So when I do have a meeting or hangout, with one or two moms, you don’t hear as many positive statements about, “Oh, I’m doing a great job*.” The good news is that you can teach yourself to notice your successes through practice. One way to practice is described below: Take 10–15 min a week to think about things you did well. There is nothing too small! Give the event a name or title, and write down exactly what happened [what you said or did; what others around you said or did]. It is particularly important that you note what you did that made it happen. Our successes don’t just happen randomly, we played a role in them. It can also be helpful to “capitalize” on this event, or purposefully “boost” the positive emotions that come from these events. Below are some suggested ways to do this: Sometimes just writing it down can be effective because it can help us to “relive” the positive event and also remember it better. Share what happened (on social media, to a friend on the phone) Celebrate what happened (do something nice for yourself, big or small, to make the event more memorable)
M	**Learn about asking for help** Folks who have recently given birth often face high expectations and a fear of judgment and shame. This can make it very difficult to ask for help. As one mom said “*Sometimes I don’t reach out when I should…And then, I start to get frustrated and not being able to take care of myself. And then, the emotions start to come…sometimes I feel like I can do everything on my own and then I can’t*.” But, you need to be aware of your limitations. Shouldering too much and putting your mind and body through lots of stress can be unhealthy for you. This “stress overload” looks different for different people. But here are some signs to look out for: **Emotional signs**: Your mood may change more than normal (becoming frustrated or agitated more easily) You may feel like you are “losing control” Difficulty calming your thoughts Feeling badly about yourself Starting to avoid other people **Physical signs**: Headaches Upset stomach If you are experiencing these things, you should consider asking for help. Going through stress for a prolonged period of time can lead to a number of negative health issues. It can be helpful to remind yourself that humans are social creatures, and we all need help sometimes. For a full expert-written article on this, see the following: https://www.psychologytoday.com/us/blog/emotional-mastery/201904/what-makes-it-so-hard-ask-help A key part of asking for help is being emotionally vulnerable, and expressing that you need others. This can be scary, but research suggests that this allows for closer relationships and a number of other positive things. See the following video, featuring Dr. Brené Brown speaking at *TEDxHouston* for more information on the power of vulnerability. This video is owned by TED, has not been modified, and is available at the following link: https://www.ted.com/talks/brene_brown_the_power_of_vulnerability?utm_campaign=tedspread&utm_medium=referral&utm_source=tedcomshare The licensing agreement for TED materials is also available here: https://creativecommons.org/licenses/by-nc-nd/3.0/legalcode EMBEDDED VIDEO Here are some suggestions that can increase comfort with being vulnerable (for more information, see the following article which these suggestions were drawn from this https://www.huffpost.com/entry/how-to-be-more-vulnerable-relationships_l_5daf65b0e4b0f34e3a7e0abd): **Find people in your life who display vulnerability**. Having an example to follow can help to build comfort with vulnerability **Take it slow.** Many people struggle to be vulnerable because they have had experiences earlier in life that have taught them it is dangerous to be vulnerable. Practicing opening up in small ways first can pave the way for opening up in larger ways later. **Keep in mind that people generally respond well to displays of vulnerability in others.** Think back to a time when someone opened up to you, you likely thought they were brave and were honored that they chose to open up to you. One last thing to keep in mind is that it is important to **set boundaries with the “helpers” in our lives**. Sometimes they may have strong opinions about how you should be parenting, and you may disagree with their opinions. Here is an expert-written article that has some tips about setting boundaries: https://www.psychologytoday.com/us/blog/the-couch/201903/everybody-needs-boundaries-6-ways-make-them-work-you
N	**Consider joining a support group** It may be helpful to spend some time around other people who are going through a similar stage of life. You might consider joining a parent support group (either in person or online). As one mom described her experience in a mom group: “ *[My baby] wouldn’t nap, I was losing my mind and I just remember crying and I was so embarrassed and I opened my eyes and half the room was crying with me. They got it. It wasn’t even like they pitied me, it was just like they knew what I was talking about and they couldn’t give me anything to fix it. But just knowing that they felt it and were with me was enough to make me breathe again*.” Below are some places to start: Online support groups from Postpartum Support international: https://www.postpartum.net/get-help/psi-online-support-meetings/ Look for parent meet-ups: https://www.meetup.com/ Mocha Moms, specifically for moms of color: https://www.mochamoms.org/i4a/pages/index.cfm?pageid=1 Group Peer Support (search specifically for parenting groups): https://grouppeersupport.org/ Search Facebook for local mom groups For parents who have had a baby in the Neonatal Intensive Care Unit (NICU) check out the organization HandtoHold: https://handtohold.org/
O	**Learn about setting a routine for baby** It can be helpful to set up routines for your baby, though it is important to also remember to be flexible and pay attention to your instinct for what your baby needs at any given time. As one mom described the helpfulness of a routine: *“Knowing that both of my children are content, and we’re prepared for the next day, and knowing that they’re feeling well, and I’m not anticipating disrupted sleep or those sort of things which, I think, I have been able to really establish through a really good routine with them. So I’m able to sort of feel that calm at the end of the day*.” Here is some information about getting your baby started on routines: https://www.babycenter.com/0_the-basics-of-baby-schedules-why-when-and-how-to-start-a-rou_3658352.bc The video below, created by Hello Doctor SA, discusses getting baby into a routine and is also available at this link: https://www.youtube.com/watch?v=yau8wEAjZgM EMBEDDED VIDEO Research suggests that sleep routines may be especially important for your baby. Below are some resources that can help with this: The videos below discuss sleeping routines for very young babies. The first was created by Centura Health and is also available at the following link: https://www.youtube.com/watch?v=mOJ4GlVNvRU. The second was created by the Mount Sinai Parenting Center and is available at the following link: https://www.youtube.com/watch?v=oU4OvpqXcJU EMBEDDED VIDEO EMBEDDED VIDEO One helpful step for establishing routines is tracking activity, or writing down your baby’s behaviors so that you can see patterns. This free app allows you to track sleep and also allows you to ask questions of experts (search in the Apple App Store or Google Play): https://www.johnsonsbaby.com/bedtime-baby-sleep-app The app Huckleberry can also help with tracking your baby’s sleep and connecting you with expert advice: https://huckleberrycare.com/ For tracking multiple behaviors try searching for the “Baby Tracker” app in the Apple Store or Google Play
P	**Get trustworthy information** Whether or not this is your first baby, you are likely going to have questions and encounter new situations that you haven’t dealt with before. It is important to have ways to get your questions answered and get the information you need. As one mom said, “*Because we as new moms, we just feel like we got it, we can handle it. We’re supposed to be able to know what we’re doing, but it’s not true. Sometimes it’s overwhelming…if you need help with something like you don’t know things, ask other people…. Don’t think because you’re asking questions that doesn’t mean you know what you’re doing*.” Below are some informational websites that are organized by topic. **Questions about the 4th trimester (first three months after having a baby):** Newmomhealth.com, Expert-written resources for folks in the postpartum period: https://newmomhealth.com/#village **Breastfeeding** La Leche League, resources for breastfeeding support https://www.llli.org/resources/ Kelly Mom, information about breastfeeding and other parenting topics: https://kellymom.com/category/about/ Happy Family, provides forum to chat with an expert for help with breastfeeding and nutrition): https://www.happyfamilyorganics.com/#chat-now **Safety**: Searchable database of expert-written safety tips: https://www.safekids.org/safetytips **Sleep**: Expert-written information https://www.babysleep.com/ **General Information** American Academy of Pediatrics Website, can search for information by age and topic: https://www.healthychildren.org/English/Pages/default.aspx For general information **about newborn care**, you can also reference Mt. Sinai Parenting Center’s Youtube channel which has videos on a range of topics: https://www.youtube.com/channel/UC7aKhPwmkYwBVz61tsBJ4Aw/videos https://newsmomsneed.marchofdimes.org/ The Motherhood Center has webinars (some available for free others for a small fee) on a number of topics: https://www.themotherhoodcenter.com/webinars See this link for educational articles **as well as a directory to locate perinatal health specialists**: https://wearerobyn.co/learn **Concerns about developmental delays**: The Center for Disease Control and Prevention has information about early intervention and how to find services near you: https://www.cdc.gov/ncbddd/actearly/parents/states.html **Note**: if you are concerned about your baby’s development, a good first step is to visit your pediatrician for a checkup. Make sure to tell the doctor what you are noticing with your baby that makes you worry. For Black birth parents, you can look into in-person perinatal safe spots providing access, connections, knowledge, and empowerment in order to “eliminate racial and class disparities in birth outcomes”: https://perinataltaskforce.com/official-perinatal-safe-spot-profiles/ You may also want to check out this website https://motherfigure.com/ which can help you to find perinatal specialists in your area.
Q	**Take a break** It is very easy to put your needs last when you have a new baby. But, taking breaks from infant care and getting out of the house are important for you and your baby. This allows for rest, which is helpful for interacting with your baby with higher energy and a more upbeat mood. Sometimes when you don’t get a break it can be hard to appreciate time with your baby. Also, especially right after giving birth, when it is a lot of work to care for baby, you can feel isolated and feel like you are losing part of your identity (your sense of who you are; this is especially true for moms who are used to working/doing lots of things outside of the house). As one mom said “*Don’t cancel out all the things that you were doing before because I know you’re busy, and you have a newborn, and you’re trying to focus on it, but try to remember some of the things that you used to like doing before you had the baby…my husband, he literally told me, “I wish you would go out with your friends [laughter].” …he was like, “No, it’s not good for you. You need to go out and socialize with people.” So try not to close off because when you close off from people, you don’t notice it, but your moods change when you get around people*.” Make a SMART goal for one thing you can do outside of the house each week. These goals should be: **S**pecific: goal should be simple, straightforward and have the details clearly outlined (When, where, what). Clearly defining your goal will help you to focus your efforts. **M**easurable: pick something with measurable progress so that you can observe the change occurring as you attain your goal. **A**ttainable: Pick something that is within reach. Taking one step at a time is a proven way to make progress towards goals. **R**elevant: Pick something you care about and are willing to work towards, this is important because the more you care, the more likely you will stick with your goal and see it through. **Ti**me-based: It is helpful to set a timeframe for your goal (within the next week I will___, within the next month I will___).
R	**Take some time to reflect** Especially when you are going through a challenging transition time, like after having a baby, it is important to know yourself, so that you know what to do to make the transition less stressful. This knowledge involves the answers to questions like: what helps me feel relaxed? What are the signs that I am getting overwhelmed? Which of my habits are helping me and which are hurting me? As one mom said “*We just have your moments when you’re down. I think it’s important to know what to do when you’re not feeling well, like to reach out for help, reach out to a friend, reach out to your mom, say, “Can you come down and take her for a little bit?” You have your moments when you’re not feeling well, but it’s having the tools to know how to get yourself out of it*.” One way to begin to build your knowledge about yourself is to “collect data.” One way to do this is to make a thought record in which you write down information about experiences that you’ve had. You can do this on a piece of paper, or even in a notepad app on your phone. To do this, take a pause at least once a day (even for five minutes!) to think back about events during that day and **note the following:** 1) what happened (what time is it, who is there, what are they doing?) 2) what were you thinking when it happened? 3) what were you feeling? 4) what did you do in response? Once you collect a couple weeks of data, you can start to see patterns in your responses, and gain a better understanding of how your emotions, thoughts, and behaviors relate to the events of your life. This gives you valuable information for planning in the future so that you “set yourself up for success” and not for low mood, anxiety, stress, or negative feelings about others in your life.
S	**Learn about resources to help you have a safe and healthy home** The environment surrounding you and your baby is important, and influences the wellbeing of your baby and yourself. Not living in stable housing or having unhealthy relationships in the household can have a major negative impact on your adjustment to having a young baby and your beliefs about yourself as a parent. If you don’t have stable housing, here is a website that can assist you: https://www.voa.org/find-housing If you don’t feel safe at home because of the individuals you live with, there are domestic violence resources available here: https://www.thehotline.org/
T	**Take care of yourself** With a new baby to care for, *your* needs (showering, food, rest, time to yourself) might take a back seat. However, it is important to take your own needs seriously. This is important not only for your wellbeing, but also for your baby’s. Taking care of yourself will help you: be a better parent (you can’t pour from an empty cup) make sure you keep a sense of your self-worth set a good example for your kids of the importance of taking care of yourself improve your physical and mental health (caregiver “burn out” has been shown to lead to mental and physical health issues) As one mom described the importance of taking care of yourself: “*Because when [you don’t have time for yourself] you sort of sink into yourself as a mom…And so you don’t parent well. You don’t cope well with things that are happening or having to put dinner on the table. It sucks… It’s like running on empty*.” While it can seem impossible to have time to yourself, even doing small things for yourself can make a big difference. Below are some ideas that were drawn from this article: https://www.verywellfamily.com/how-to-practice-self-care-as-a-new-mom-4771779 **Get into a hydration routine**. Fill a big water bottle up in the morning and bring it with you throughout the day. You could also put lemon, lime, or other flavoring in it. **Give yourself permission to rest**. For at least one of your baby’s naps, let yourself not do anything. There are a lot of expectations on new moms, but we all need time to just breathe. **Put some music on while you clean**. Adding some extra motion (dancing, picking up your pace) while you clean can raise your heart rate and work in a little extra exercise into your day. Also, exercising can be an important way of taking care of yourself. It can improve mental and physical health, and can provide you with a recharging break away from taking care of the baby. But it can raise some questions: *How do I start exercising after childbirth?* It’s important to take it slow and ease back into exercise after having a baby. You should ask your doctor about exercising in the first few months after birth. Read this article for some tips about easing back in: https://www.parents.com/pregnancy/my-body/postpartum/postpartum-exercise/ *What kind of exercise should I do?* Walking is a great, gentle cardiovascular exercise. Plus the benefit of getting outside is also good for your mental health. There are also free online videos that you can do which incorporate your baby into the exercise, see below for some examples: https://www.thebump.com/a/exercises-to-do-with-baby https://www.parents.com/parenting/moms/healthy-mom/mommy-and-me-workout/ The video below, created by BodyFit by Amy, has an 18 min “mommy and me” workout. It is also available at the following link: https://www.youtube.com/watch?v=Ni9MxZpCHbk EMBEDDED VIDEO Fitness blender has free customizable videos, though they are not postpartum-specific, so listen to your body as you ease back in https://www.fitnessblender.com/videos Try Yoga, it’s good to build your body’s strength and also good for gaining mindfulness skills (learning to focus on the present movement) that can help with the health of the full mind-body system Yoga with Adrienne Youtube Channel https://www.youtube.com/user/yogawithadriene SarahBeth Yoga Youtube Channel, has some videos that are postpartum specific! https://www.youtube.com/user/SarahBethShow
U	**Make sleep a priority** You have probably heard the advice to “sleep when the baby sleeps”. This is likely easier said than done, especially during the day when it may not be easy to fall asleep and you may want to get other things done. However, it is important to try to sleep when the baby sleeps at night. In addition to reducing feelings of exhaustion and fatigue, sleep is important for our health in the following ways (taken from this article https://www.nhs.uk/live-well/sleep-and-tiredness/why-lack-of-sleep-is-bad-for-your-health/): Sleep boosts your immune system which helps fight off infections and sickness Sleep helps to regulate your hunger and keep you from overeating Better sleep reduces the risk of mental health conditions like depression and anxiety Sleep is good for heart health If you can take advantage of pockets of time throughout the day to catch up on some sleep, do it! It is a cornerstone of health. If you find yourself struggling to fall asleep at night when the baby is sleeping (you lay in bed for hours, or have issues staying asleep even when you aren’t awoken by baby), you may be experiencing insomnia. The good news is that there are lots of effective ways to tackle this problem! A good first step is talking to your doctor, but the tips below can be helpful as well: Set yourself up for good sleep: Don’t do anything but sleep and have sex in your bed. The more activities you do in bed (read, work, browse on your laptop, watch TV), the more you will associate your bed with activity. Try to keep your bedroom cool and dark. Consider using white noise to block out sounds around you for example you can download the following app onto your smartphone: https://apps.apple.com/us/app/sleep-aid-fan-white-noise/id1125321779 Turn the alarm clock around to avoid “clock watching” Try listening to a relaxing audio recording when you get into bed. For example, you might try listening to one of “The Honest Guys” free Youtube videos, many of which are made to help people relax before bed. https://www.youtube.com/channel/UC4jWo5kiyOCt4PnvF4jbaLg If baby’s sleep is impacting your ability to get rest, here is a resource of expert-written information on baby sleep to give you a place to start working through questions or concerns you may have about baby’s sleep: https://www.babysleep.com/ Even if you can’t fall completely asleep, just taking a few minutes to rest (perhaps reading a book or just sitting in a comfortable chair) can be helpful.
V	**Limit social media scrolling** New moms have a lot on their plates, and it is especially hard to be a new mom in our culture. Not only does society blame mothers for any struggles or issues experienced by their children, but we are also surrounded by “perfect” images on moms on TV and the Internet. It can be hard to not feel judged, or to not judge yourself, when you are in a culture like that. As one mom said “*I think that on Instagram those moms who are like, “I love my baby so much. They’re beautiful.” And I loved her but in the beginning, I personally, didn’t necessarily feel like—I mean, I loved her. I cried when I saw her. She was beautiful, but I was also exhausted and semi miserable about everything. So seeing other people say that they loved their baby and they loved their husband. That’s a huge one. Saying they loved their partner. I’m like, “You can’t, he’s a disaster. He doesn’t do anything right.” I think that made me feel really bad. Like, I’m the only one who hates my husband right now*.” But, we can set ourselves up for less comparison, by **limiting social media use**, especially when you are using it to look at other people’s profiles. Research suggests that this makes us very focused on comparing ourselves to others and has been linked to low mood and other negative mental health impacts. There are also apps for your smartphone that you can download that can help you track time spent on social media and help limit your usage.
W	**Learn about some tips for effective time management** You have a lot on your plate and have to do a lot of thinking and planning in order to make sure everything on your to-do list gets done. While you can’t control everything, below are some articles with tips that might help you to organize your days: https://www.busybudgeter.com/time-management-tips-for-busy-moms/ https://www.psychologytoday.com/us/blog/understand-other-people/201507/developing-time-management-skills https://www.psychologytoday.com/us/blog/between-you-and-me/201912/need-make-plan-try-starting-the-end
X	**Find someone to talk to** Find individuals in your life that you can talk to about your feelings and experiences. This is important as you go through the postpartum transition. Previous research has shown that moms really benefit from having this kind of support. As one mom said “*I think someone to talk to is the biggest one. Because I just—I don’t know. I had so many doubts and whether I was doing the right thing or not or trying to figure out the right thing to do for a lot of different scenarios. So yeah, just helped me to be able to talk about that to somebody*.” But, it can be hard if you don’t have folks nearby that you feel comfortable opening up to. However, there are some online, free resources that can provide this kind of support. 7cups is a service where you can chat online for free, https://www.7cups.com/
Y	**Learn about community support resources** It is an incredible amount of work to have a new baby. Society puts a lot of expectations on parents to manage all the responsibilities of their new baby while also continuing along with all of their other responsibilities. Many would argue that this is too much for one person! But, getting help with childcare and managing the home can also be expensive. However, in many places in the US there are organizations called “Mutual Aid” groups, which provide community support to those around them. This can involve help with shopping and food delivery, childcare, or other services. Check out the links below to see what is available in your community! Mutual Aid Hub listing of community self-supported projects https://www.mutualaidhub.org/ Idealist listing of mutual aid/community self-supported projects https://www.idealist.org/en/groups?q=&searchMode=true If you could specifically benefit from help with childcare, search for free or reduced cost care services near you (enter your Zipcode and then select “care” tab at top): https://www.auntbertha.com/ There are also tools that can help you get the most support from your network of family and friends: https://www.mealtrain.com can help you to organize “meal trains” (when folks in your life offer to bring you a meal) https://lotsahelpinghands.com/ can help you to let family and friends know how to best help you
Z	**Learn the “language” of your baby** Babies can’t tell you what they need in words, but they communicate with you in other ways. Being able to understand the “language” of your baby is key to understanding what they need. It can be very upsetting to not understand what your baby is communicating, especially when they are crying. Below are some educational resources to help you to learn the ‘language’ of your infant. Learn about what things affect your baby’s temperament, so you can better understand how your child ‘ticks’: https://www.zerotothree.org/resources/159-temperament-what-makes-your-child-tick Learn about how your response to your infant works to develop their attachment: https://www.zerotothree.org/resources/230-responsive-care-nurturing-a-strong-attachment-through-everyday-moments Here are some resources to help you to better understand your baby’s cues: The video below was created by Bundoo and discusses six different baby cries and what they mean. It is also available at this link: https://www.youtube.com/watch?v=Oyur-q0gGAs EMBEDDED VIDEO Here is a ‘playlist’ of videos about baby behavior by mn health: https://www.youtube.com/watch?v=lLUJV0QLAiY&list=PLnv1INVkmxmtYaMmowS5oBHnbgmps7Ai1 http://csefel.vanderbilt.edu/documents/reading_cues.pdf https://www.childrensmn.org/educationmaterials/childrensmn/article/15276/infant-behavior-cues/ https://www.fhs.gov.hk/english/health_info/child/30119.pdf https://www.fhs.gov.hk/english/health_info/child/13041.html
a	**Know your rights as an employee** If you or your partner is working, the way you/your partner are treated by your employers will have a big effect on your postpartum adjustment. It is important to know your rights when it comes to working during pregnancy and postpartum. Here are is a website that can help you learn those rights: https://www.familyeducation.com/pregnancy/maternity-leave/maternity-leave-laws-what-are-your-rights Here is a government link in case you have questions or want to learn more: https://www.womenshealth.gov/pregnancy/youre-pregnant-now-what/know-your-pregnancy-rights If you require legal help related to your work, here is some information for getting low cost legal help: https://www.americanbar.org/groups/legal_services/flh-home/flh-free-legal-help/ It may also be helpful to check out http://www.thefifthtrimester.com/for-moms-2, which has information about transitioning back to work (scroll down for resources).
B	**Address your physical pain and discomfort** It is common to experience physical pain and discomfort during the postpartum period. Below are some resources to help address this, **but it is also important to discuss your concerns with your medical provider.** Some of your pain or discomfort may be due to the physical recovery from pregnancy and birth. Here are some websites that provide information about physical recovery from pregnancy and birth: https://newmomhealth.com/ https://www.mayoclinic.org/healthy-lifestyle/labor-and-delivery/basics/postpartum-care/hlv-20049465 (scroll down for articles covering related topics in more detail) Your pain or discomfort may also be due to the repetitive strain/overuse of body during the postpartum period (also called the “ergonomic demands” of baby care). Here are some websites that may be able to help with this kind of discomfort: Baby wearing: https://www.mother.ly/lifestyle/ergonomics-for-moms-babywearing Breast feeding: https://www.mother.ly/lifestyle/ergonomics-for-moms-breastfeeding-positions Daily life: https://www.babyproductsmom.com/2016/03/ergonomic-nursery-tips-tricks-make-parenting-easieron/ If you are experiencing pain or discomfort surrounding urination or sexual intercourse, it may be helpful to look into some specific information about the pelvic floor (the muscles that support your womb, bladder, and bowel). Here are some places to start: http://functionalpelvis.com/ https://healthcare.utah.edu/womenshealth/gynecology/postpartum-pelvic-floor-disorders.php Specialists, like physical or occupational therapists, may also be helpful in relieving physical pain and discomfort. Here are some directories to help you find specialists near you: https://motherfigure.com/ https://wearerobyn.co/

[*if individuals endorse all factors as a strength*] You seem to be adjusting very well to life after having a baby. That is great! As you move forward, continue to be kind with yourself and flexible about your expectations. Also, don’t forget how important it is to take care of you! It can be easy to lose sight of this when you have a little one. Your ability to take a break and take care of yourself is good both for you and your baby. [*the following language appeared after the curated list of resources*]

If you are experiencing any thoughts of hurting yourself or anyone else, **please contact**:

National Hopeline Network

1-800-SUICIDE (1-800-784-2433)
www.hopeline.com

National Strategy for Suicide Prevention: Lifeline

1-800-273-TALK (1-800-273-8255)
www.mentalhealth.samhsa.gov/suicideprevention

Text HOME to 741741 to connect with a Crisis Counselor, Free 24/7 support at your fingertips US and Canada: text 741741

If you are experiencing the urge to hurt yourself or anyone else, please call 911 or proceed to your nearest emergency room.

## Appendix C. Mapping of Resources to Checklist Items

**Checklist Item**	**Resource Recommendations**
1. When things don’t go well, I don’t blame myself.	A “Question self-blame” B “Increase self-compassion” C “Consider seeking mental health support”
2. When trying new things, I do all of the following: - I am patient with myself and don’t expect myself to get things right away - I feel comfortable pushing myself beyond my comfort zone - I can be flexible with my plans and expectations.	B “Increase self-compassion” C “Consider seeking mental health support” D “Use positive affirmations” E “Get some distance from your thoughts”
3. My baby and I are bonding.	F “Learn about bonding with your baby”
4. Most of the time my baby’s crying doesn’t overwhelm me.	G “Calm your baby and yourself through crying spells”
5. Most of the time I am able to calm myself down when I have strong emotions (such as stress, worries, anger) or distressing thoughts.	C “Consider seeking mental health support” E “Get some distance from your thoughts” H “Gain some tools to help you manage strong emotions”
6. I have people in my life that help to make sure I’m taking care of myself. For example, people in my life check-in with me about taking care of myself, or they help me to take care of myself by doing things like watching the baby.	I “Consider a postpartum doula”
7. I am able to get the materials I need to take care of the baby and myself (such as diapers and food).	J “Learn about the resources around you”
8. I am getting more experienced with taking care of my baby.	K “Learn about baby care”
9. When I do things well, I am able to give myself credit for it.	L “Increase the power of positive emotions”
10. *If you have people in your life who help and support you*: when I am talking to people in my life who help or support me, I do all of the following: - I can be open and honest - I can trust people to help me - I can ask for help and take help when it is offered	C “Consider seeking mental health support” M “Learn about asking for help” N “Consider joining a support group”
11. I have been able to get my baby into a routine.	O “Learn about setting a routine for baby”
12. When I have a question about parenting or early motherhood, I know how to get an answer that I trust.	I “Consider a postpartum doula” N “Consider joining a support group” P “Get trustworthy information”
13. I am involved in activities outside of the house (such as friendships, hobbies, professional or volunteer work).	Q “Take a break”
14. I know myself (when I need to ask for help, what I need, what makes me feel better when I feel down, worried, or stressed).	C “Consider seeking mental health support” R “Take some time to reflect”
15. My home environment is safe and stable.	S “Learn about resources to help you have a safe and healthy home”
16. I know that taking care of myself is important, and guilt does not get in the way of taking care of my own needs.	E “Get some distance from your thoughts” T “Take care of yourself”
17. I am getting as much sleep as I can.	U “Make sleep a priority”
18. When thinking about social pressures, I don’t do any of the following: - feel like I have to reach societal expectations - worry about other people’s judgments of me - compare myself to others.	E “Get some distance from your thoughts” V “Limit social media scrolling”
19. I plan and manage time well.	W “Learn some tips for effective time management”
20. I have people in my life that I can talk to openly and honestly about my feelings and experiences.	N “Consider joining a support group” X “Find someone to talk to”
21. I have people to help me with childcare and household management (cooking, cleaning, shopping, etc.).	I “Consider a postpartum doula” Y “Learn about community support resources”
22. I understand what my baby is communicating to me (through their cries, body language, etc.).	Z “Learn the ‘language’ of your baby”
23. *If you or your co-parent are currently working*: my workplace/my partner’s workplace has been understanding and flexible since I have had my baby.	A “Know your rights as an employee”
24. I am not experiencing physical pain or discomfort.	B “Address your physical pain and discomfort”

## Appendix D

**Table A1:** Report of resource offering and use.

	Total (*n* = 124) ^a^
**Average Percentage of Reported Use Across all Resources**	24.29
**Resource 1: Address Physical Pain and Discomfort**	***n* (%)**
Number of participants to whom the resource was offered	40 (32.3)
Number of participants offered the resource who reported using the resource or the intention to use the resource	12 (30)
**Resource 2: Calm your baby and yourself through crying spells**	
Number of participants to whom the resource was offered	72 (58.1)
Number of participants offered the resource who reported using the resource or the intention to use the resource	25 (34.72)
**Resource 3: Consider a postpartum doula**	
Number of participants to whom the resource was offered	93 (75)
Number of participants offered the resource who reported using the resource or the intention to use the resource	5 (5.38)
**Resource 4: Consider joining a support group**	
Number of participants to whom the resource was offered	91 (73.4)
Number of participants offered the resource who reported using the resource or the intention to use the resource	19 (20.9)
**Resource 5: Consider seeking professional mental health support**	
Number of participants to whom the resource was offered	122 (98.4)
Number of participants offered the resource who reported using the resource or the intention to use the resource	29 (23.77)
**Resource 6: Find someone to talk to**	
Number of participants to whom the resource was offered	39 (31.45)
Number of participants offered the resource who reported using the resource or the intention to use the resource	6 (15.38)
**Resource 7: Gain some tools to help you manage strong emotions**	
Number of participants to whom the resource was offered	89 (71.8)
Number of participants offered the resource who reported using the resource or the intention to use the resource	24 (26.3)
**Resource 8: Get some distance from your thoughts**	
Number of participants to whom the resource was offered	122 (98.4)
Number of participants offered the resource who reported using the resource or the intention to use the resource	25 (20.16)
**Resource 9: Get trustworthy information**	
Number of participants to whom the resource was offered	36 (29)
Number of participants offered the resource who reported using the resource or the intention to use the resource	5 (13.9)
**Resource 10: Increase self-compassion**	
Number of participants to whom the resource was offered	110 (88.7)
Number of participants offered the resource who reported using the resource or the intention to use the resource	43 (39.09)
**Resource 11: Increase the power of positive emotions**	
Number of participants to whom the resource was offered	52 (41.9)
Number of participants offered the resource who reported using the resource or the intention to use the resource	8 (15.38)
**Resource 12: Know your rights as an employee**	
Number of participants to whom the resource was offered	49 (39.5)
Number of participants offered the resource who reported using the resource or the intention to use the resource	6 (12.24)
**Resource 13: Learn about asking for help**	
Number of participants to whom the resource was offered	72 (58.1)
Number of participants offered the resource who reported using the resource or the intention to use the resource	20 (27.78)
**Resource 14: Learn about baby care**	
Number of participants to whom the resource was offered	6 (4.8)
Number of participants offered the resource who reported using the resource or the intention to use the resource	0 (0)
**Resource 15: Learn about bonding with your baby**	
Number of participants to whom the resource was offered	21 (16.94)
Number of participants offered the resource who reported using the resource or the intention to use the resource	4 (19.05)
**Resource 16: Learn about community support resources**	
Number of participants to whom the resource was offered	67 (54.03)
Number of participants offered the resource who reported using the resource or the intention to use the resource	12 (17.91)
**Resource 17: Learn about resources to help you have a safe and healthy home**	
Number of participants to whom the resource was offered	8 (6.45)
Number of participants offered the resource who reported using the resource or the intention to use the resource	1 (0.81)
**Resource 18: Learn about setting a routine for baby**	
Number of participants to whom the resource was offered	62 (50)
Number of participants offered the resource who reported using the resource or the intention to use the resource	21 (33.87)
**Resource 19: Learn about some tips for effective time management**	
Number of participants to whom the resource was offered	68 (54.84)
Number of participants offered the resource who reported using the resource or the intention to use the resource	20 (29.41)
**Resource 20: Learn about the resources around you**	
Number of participants to whom the resource was offered	5 (4.01)
Number of participants offered the resource who reported using the resource or the intention to use the resource	0 (0)
**Resource 21: Learn the ‘language’ of your baby**	
Number of participants to whom the resource was offered	46 (37.1)
Number of participants offered the resource who reported using the resource or the intention to use the resource	19 (41.3)
**Resource 22: Limit social media scrolling**	
Number of participants to whom the resource was offered	78 (62.9)
Number of participants offered the resource who reported using the resource or the intention to use the resource	29 (37.18)
**Resource 23: Make sleep a priority**	
Number of participants to whom the resource was offered	58 (46.77)
Number of participants offered the resource who reported using the resource or the intention to use the resource	22 (37.93)
**Resource 24: Question self-blame**	
Number of participants to whom the resource was offered	87 (70.16)
Number of participants offered the resource who reported using the resource or the intention to use the resource	22 (25.29)
**Resource 25: Take a break**	
Number of participants to whom the resource was offered	92 (74.19)
Number of participants offered the resource who reported using the resource or the intention to use the resource	40 (43.48)
**Resource 26: Take care of yourself**	
Number of participants to whom the resource was offered	98 (79.03)
Number of participants offered the resource who reported using the resource or the intention to use the resource	44 (44.9)
**Resource 27: Take some time to reflect**	
Number of participants to whom the resource was offered	81 (65.32)
Number of participants offered the resource who reported using the resource or the intention to use the resource	18 (22.22)
**Resource 28: Use positive affirmation**	
Number of participants to whom the resource was offered	98 (79.03)
Number of participants offered the resource who reported using the resource or the intention to use the resource	41 (41.84)

^a^ 124 participants were used in outcome analysis as one individual responded to The Postpartum Toolkit Checklist endorsing all strengths and thus was not presented with resources.

## Appendix E

**Table A2:** Frequency counts of responses to The Postpartum Toolkit checklist items.

	Total (*n* = 125)
**1. When things don’t go well, I don’t blame myself.**	***n (%)***
This is not an issue for me.	38 (30.4)
This is going ok for me, but could be better.	66 (52.8)
I’d like help with this.	21 (16.8)
**2. When trying new things, I do all of the following:** **I am patient with myself and don’t expect myself to get things right away** **I feel comfortable pushing myself beyond my comfort zone** **I can be flexible with my plans and expectations.**	
This is a strength for me.	27 (21.6)
This is going ok for me, but could be better.	77 (61.6)
I’d like help with this.	21 (16.8)
**3. My baby and I are bonding.**	
This is going well for me.	104 (83.2)
This is going ok for me, but could be better.	19 (15.2)
I’d like help with this.	2 (1.6)
**4. Most of the time my baby’s crying doesn’t overwhelm me.**	
This is a strength for me.	53 (42.4)
This is going ok for me, but could be better.	60 (48)
I’d like help with this.	12 (9.6)
**5. Most of the time I am able to calm myself down when I have strong emotions (such as stress, worries, anger) or distressing thoughts.**	
This is a strength for me.	36 (28.8)
This is going ok for me, but could be better.	67 (53.6)
I’d like help with this.	22 (17.6)
**6. I have people in my life that help to make sure I’m taking care of myself. For example, people in my life check-in with me about taking care of myself, or they help me to take care of myself by doing things like watching the baby.**	
This is going well for me.	65 (52)
This is going ok for me, but could be better.	46 (36.8)
I’d like help with this.	14 (11.2)
**7. I am able to get the materials I need to take care of the baby and myself (such as diapers and food).**	
This is not an issue for me.	120 (96)
This is going ok for me, but could be better.	4 (3.2)
I’d like help with this.	1 (.8)
**8. I am getting more experienced with taking care of my baby.**	
This is going well for me.	119 (95.2)
This is going ok for me, but could be better.	6 (4.8)
I’d like help with this.	0
**9. When I do things well, I am able to give myself credit for it.**	
This is a strength for me.	73 (58.4)
This is going ok for me, but could be better.	48 (38.4)
I’d like help with this.	4 (3.2)
10. *If you have people in your life who help and support you***: when I am talking to people in my life who help or support me, I do all of the following:** **I can be open and honest** **I can trust people to help me** **I can ask for help and take help when it is offered**	
This is a strength for me.	53 (42.2)
This is going ok for me, but could be better.	61 (48.8)
I’d like help with this.	11 (8.8)
**11. I have been able to get my baby into a routine.**	
This is going well for me.	63 (50.4)
This is going ok for me, but could be better.	49 (39.2)
I’d like help with this.	13 (10.4)
**12. When I have a question about parenting or early motherhood, I know how to get an answer that I trust.**	
This is going well for me.	89 (71.2)
This is going ok for me, but could be better.	32 (25.6)
I’d like help with this.	4 (3.2)
**13. I am involved in activities outside of the house (such as friendships, hobbies, professional or volunteer work).**	
This is going well for me.	33 (26.4)
This is going ok for me, but could be better	69 (55.2)
I’d like help with this.	23 (18.4)
**14. I know myself (when I need to ask for help, what I need, what makes me feel better when I feel down, worried, or stressed).**	
This is a strength for me.	44 (35.2)
This is going ok for me, but could be better.	64 (51.2)
I’d like help with this.	17 (13.6)
**15. My home environment is safe and stable.** This is not an issue for me.	117 (93.6)
This is going ok for me, but could be better.	7 (5.6)
I’d like help with this.	1 (.8)
**16. I know that taking care of myself is important, and guilt does not get in the way of taking care of my own needs.**	
This is a strength for me.	27 (21.6)
This is going ok for me, but could be better.	71 (56.8)
I’d like help with this.	27 (21.6)
**17. I am able to sleep at night when my baby is sleeping.**	
This is going well for me.	67 (53.6)
This is going ok for me, but could be better.	45 (36)
I’d like help with this.	13 (10.4)
**18. When thinking about social pressures, I don’t do any of the following:** **feel like I have to reach the expectations of others** **worry about other people’s judgments of me** **compare myself to others.**	
This is a strength for me.	47 (37.6)
This is going ok for me, but could be better.	64 (51.2)
I’d like help with this.	14 (11.2)
**19. I plan and manage time well.**	
This is a strength for me.	57 (45.6)
This is going ok for me, but could be better.	51 (40.8)
I’d like help with this.	17 (13.6)
**20. I have people in my life that I can talk to openly and honestly about my feelings and experiences.**	
This is not an issue for me.	86 (68.8)
This is going ok for me, but could be better.	33 (26.4)
I’d like help with this.	6 (4.8)
**21. I have people to help me with childcare and household management (cooking, cleaning, shopping, etc.).**	
This is not an issue for me.	58 (46.4)
This is going ok for me, but could be better.	50 (40)
I’d like help with this.	17 (13.6)
**22. I understand what my baby is trying to tell me (through their cries, body language, etc.).**	
This is a strength for me.	79 (63.2)
This is going ok for me, but could be better.	43 (34.3)
I’d like help with this.	3 (2.4)
**23. *If you or your co-parent are currently working*: my workplace/my partner’s workplace has been understanding and flexible since I have had my baby.**	
This is not an issue for me.	76 (60.8)
This is going ok for me, but could be better.	42 (33.6)
I’d like help with this.	7 (5.6)
**24. I am not experiencing physical pain or discomfort.**	
This is not an issue for me.	85 (68)
This is going ok for me, but could be better.	29 (23.2)
I’d like help with this.	11 (8.8)

## Appendix F. Item Level Average for the BIMF

	**Total (*n* = 124)**
**1. I am a good mother.**	**M ± SD**
Baseline	5.06 ± 0.94
Follow up	5.15 ± 0.91
**2. I feel rested.**	
Baseline	2.53 ± 1.6
Follow up	2.9 ± 1.66
**3. I am comfortable with the way I’ve** **chosen to feed my baby (either bottle or** **breast, or both).**	
Baseline	5.21 ± 1.12
Follow up	5.4 ± 1.1
**4. My baby and I understand each other.**	
Baseline	4.92 ± 0.98
Follow up	5.03 ± 0.93
**5. I am able to relax and enjoy time with** **my baby.**	
Baseline	4.41 ± 1.28
Follow up	4.73 ± 1.04
**6. There are people in my life that I can** **trust to care for my baby when I need a** **break.**	
Baseline	4.91 ± 1.36
Follow up	5.91 ± 1.34
**7. I am comfortable allowing a trusted** **friend or relative to care for my baby (can include baby’s father or partner).**	
Baseline	5.25 ± 1.06
Follow up	5.31 ± 1.08
**8. I am getting enough adult interaction.**	
Baseline	3.1 ± 1.85
Follow up	3.73 ± 1.62
**9. I am getting enough encouragement** **from other people.**	
Baseline	4.05 ± 1.55
Follow up	4.35 ± 1.28
**10. I trust my own feelings (instincts)** **when it comes to taking care of my baby.**	
Baseline	4.9 ± 1.08
Follow up	5.06 ± 0.88
**11. I take a little time each week to do** **something for myself.**	
Baseline	2.96 ± 1.81
Follow up	3.4 ± 1.68
**12. I am taking good care of my baby’s** **physical needs (feedings, changing** **diapers, doctor’s appointments).**	
Baseline	5.77 ± 0.44
Follow up	5.76 ± 0.45
**13. I am taking good care of my physical** **needs (eating, showering, etc.)**	
Baseline	3.81 ± 1.56
Follow up	4.11 ± 1.54
**14. I make good decisions about my** **baby’s health and well being.**	
Baseline	5.5 ± 0.67
Follow up	5.59 ± 0.59
**15. My baby and I are getting into a** **routine.**	
Baseline	4.91 ± 1.1
Follow up	5.23 ± 0.89
**16. I worry about how other people judge me (as a mother).**	
Baseline	3.21 ± 1.81
Follow up	3.36 ± 1.84
**17. I am able to take care of my baby and my other responsibilities.**	
Baseline	3.81 ± 1.56
Follow up	4.08 ± 1.31
**18. Anxiety or worry often interferes with my mothering ability.**	
Baseline	3.36 ± 1.65
Follow up	3.69 ± 1.6
**19. As time goes on, I am getting better at taking care of my baby.**	
Baseline	5.3 ± 0.8
Follow up	5.44 ± 0.67
**20. I am satisfied with the job I am doing as a new mother.**	
Baseline	4.9 ± 0.93
Follow up	5.15 ± 0.82
